# Mixed-lineage leukemia protein 2 suppresses ciliary assembly by the modulation of actin dynamics and vesicle transport

**DOI:** 10.1038/s41421-019-0100-3

**Published:** 2019-06-25

**Authors:** Yang Yang, Huijie Hao, Xiaofan Wu, Song Guo, Yang Liu, Jie Ran, Te Li, Dengwen Li, Min Liu, Jun Zhou

**Affiliations:** 1 State Key Laboratory of Medicinal Chemical Biology, Key Laboratory of Bioactive Materials of the Ministry of Education, Tianjin Key Laboratory of Protein Science, College of Life Sciences, Nankai University, Tianjin, 300071 China; 2 Shandong Provincial Key Laboratory of Animal Resistance Biology, Collaborative Innovation Center of Cell Biology in Universities of Shandong, Institute of Biomedical Sciences, College of Life Sciences, Shandong Normal University, Jinan, Shandong 250014 China

**Keywords:** Cilia, Actin

## Abstract

Primary cilia are critically involved in the coordination of diverse signaling pathways and ciliary defects are associated with a variety of human diseases. The past decades have witnessed great progress in the core machinery orchestrating ciliary assembly. However, the upstream epigenetic cues that direct ciliogenesis remain elusive. Herein, we demonstrate that mixed-lineage leukemia protein 2 (MLL2), a histone methyltransferase, plays a negative role in ciliogenesis. RNA-sequencing analysis reveals that the expression of five actin-associated proteins is significantly downregulated in MLL2-depleted cells. Overexpression of these proteins partially rescues ciliary abnormality elicited by MLL2 depletion. Our data also show that actin dynamics is remarkably changed in MLL2-depleted cells, resulting in the impairment of cell adhesion, spreading, and motility. In addition, MLL2 depletion promotes ciliary vesicle trafficking to the basal body in an actin-related manner. Together, these results reveal that MLL2 inhibits ciliogenesis by modulating actin dynamics and vesicle transport, and suggest that alteration of MLL2 may contribute to the pathogenesis of cilium-associated diseases.

## Introduction

Primary cilia are microtubule-based structures that protrude from the cell surface of most mammalian cells and provide a platform for the coordination of many signaling pathways. Dysfunction of cilia has been linked to a number of human diseases, collectively termed ciliopathies; examples include polycystic kidney disease, retinal degeneration, and obesity^1,2^. Recent studies have indicated that defects in cilia are also associated with congenital heart disease (CHD)^3^. Several cilium-mediated pathways have been shown to play essential roles in regulating heart development and may contribute to the pathogenesis of CHD^4^. Heart defects are also observed in several ciliopathy syndromes, such as Alström syndrome and Bardet–Biedl syndrome^5,6^.

A number of studies have implicated filamentous actin (F-actin) in ciliary regulation. For example, the flagella of crane fly sperm have F-actin-like structures that bind heavy meromyosin^7^ and the connecting cilia of photoreceptors contain F-actin that plays an important role in outer segment morphogenesis^8–10^. In addition, actin and actin-associated proteins have been identified as ciliary components in proteomic studies^11,12^, and glial actin has been shown to regulate neuronal ciliogenesis^13^. F-actin has been demonstrated to exist in the primary cilia of inner medullary collecting duct cells, where it regulates the corralling of G protein-coupled receptors^14^. Recent studies have also shown that F-actin mediates ectosome release from the ciliary tip^15,16^, and that the actin-based motor protein myosin 5A (MYO5A) regulates ciliogenesis primarily by mediating the transport of preciliary vesicles to the mother centriole^11,17,18^.

Interestingly, branched F-actin, which is nucleated by the actin-related protein 2/3 (Arp2/3) complex and mainly distributed in the cell cortex, appears to inhibit ciliary formation. For example, depletion of Arp3 or a number of other actin-associated proteins promotes ciliogenesis^19,20^. Expression of miR-129-3p induces ciliogenesis and increases ciliary length through concomitant downregulation of four positive regulators of F-actin branching^21^. In addition, cortactin and missing-in-metastasis regulate ciliogenesis by regulating the polymerization of branched F-actin^22,23^. Branched F-actin may provide constricting forces that inhibit the transport of ciliary vesicles, including those positive for Rab8 and Rab11, small GTPases critically involved in membrane trafficking^24–26^.

Mixed-lineage leukemia protein 2 (MLL2), which is also named histone-lysine *N*-methyltransferase 2D, plays a critical role in regulating gene transcription^27^. In particular, it targets histone 3 lysine 4 (H3K4) and methylation of this residue serves as a marker for gene activation^28^. Patients with CHD frequently possess gene mutations for proteins involved in the regulation of H3K4 methylation, including MLL2^29^, but the precise role of MLL2 in the development of heart diseases is largely unknown. Herein, we report that MLL2 negatively regulates ciliogenesis, and that several actin-associated proteins were significantly downregulated by MLL2 depletion; overexpression of these proteins could partially rescue the ciliary abnormality in MLL2-knockdown cells. We also demonstrate that actin dynamics was impaired in MLL2-depleted cells, and that MLL2 influences ciliogenesis through a role in vesicle trafficking. These findings reveal that MLL2 ablation stimulates abnormal ciliary assembly, suggesting a potential mechanism for the pathogenic effects of MLL2 mutations in CHD.

## Results

### MLL2 is a negative regulator of ciliogenesis

**Fig. 1:**
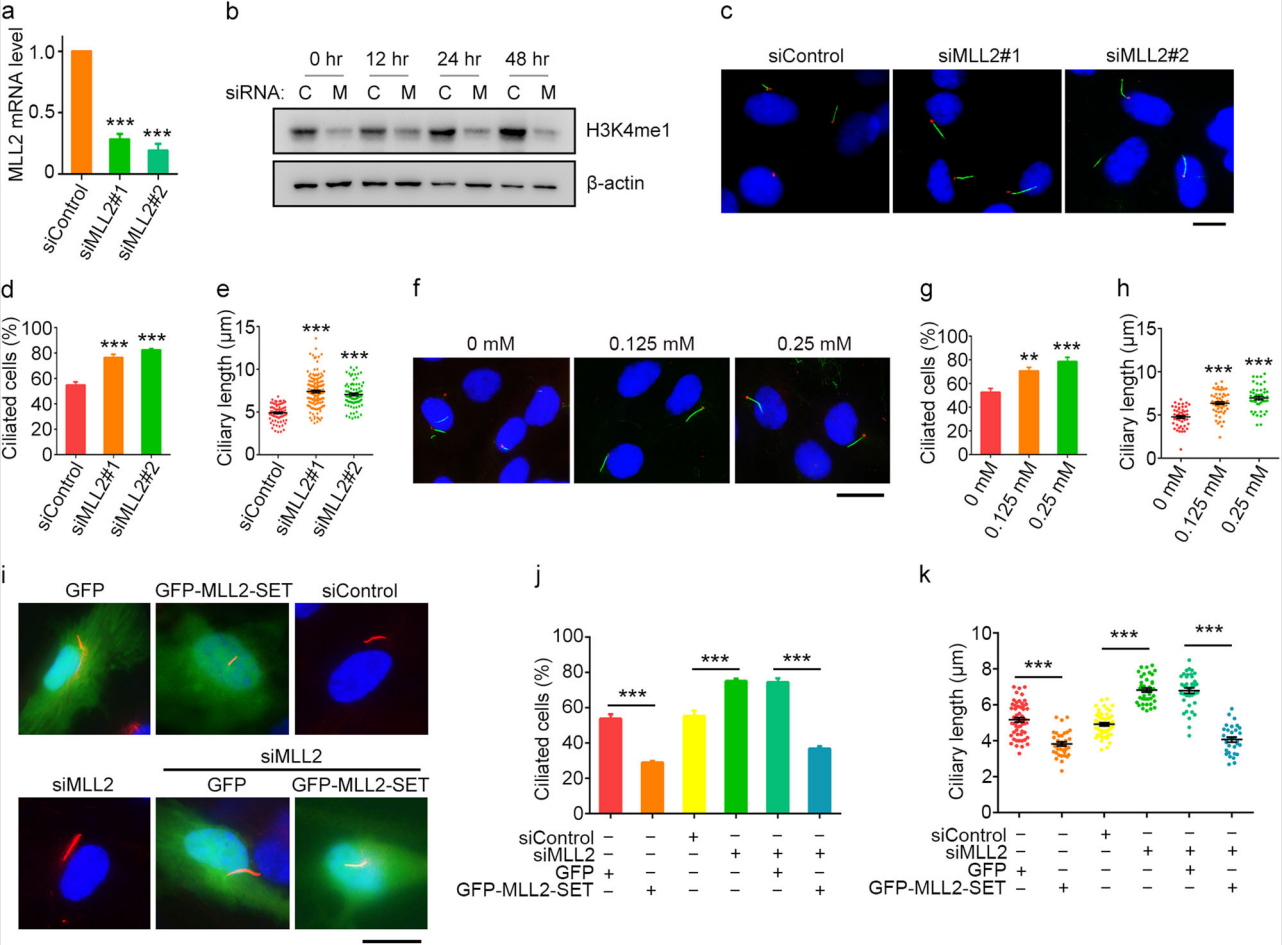
Depletion of MLL2 expression or inhibition of its activity stimulates ciliogenesis. **a** Quantitative RT-PCR analysis of MLL2 mRNA expression in RPE-1 cells transfected with control or MLL2 siRNAs and serum-starved for 48 h. **b** Immunoblot analysis of H3K4me1 and β-actin levels in RPE-1 cells transfected with control (C) or MLL2 (M) siRNAs and serum-starved for 0, 12, 24, and 48 h. **c**–**e** Immunofluorescence images (**c**), percentage of ciliated cells (**d**, *n* = 200), and ciliary length (**e**, *n* = 80) for RPE-1 cells transfected with control or MLL2 siRNAs, serum-starved for 48 h, and stained with antibodies against γ-tubulin (red) and acetylated α-tubulin (green), and the DNA dye DAPI (blue). Scale bar, 10 µm. **f**–**h** Immunofluorescence images (**f**), percentage of ciliated cells (**g**, *n* = 200), and ciliary length (**h**, *n* = 50) for RPE-1 cells treated with the indicated concentrations of MTA, serum-starved for 48 h, and stained with antibodies against γ-tubulin (red) and acetylated α-tubulin (green), and DAPI (blue). Scale bar, 10 µm. **i**–**k** Immunofluorescence images (**i**), percentage of ciliated cells (**j**, *n* = 100), and ciliary length (**k**, *n* = 30) for RPE-1 cells transfected with the indicated siRNAs and plasmids, followed by serum starvation and staining with acetylated α-tubulin antibodies (red) and DAPI (blue). Scale bar, 10 µm. **P < 0.01; ***P < 0.001. Error bars indicate SEM

To investigate the potential role of MLL2 in ciliogenesis, we examined the effects of MLL2 depletion on the formation of primary cilia induced by serum starvation in RPE-1 retinal pigment epithelial cells. Quantitative reverse transcription PCR (RT-PCR) revealed that MLL2 small interfering RNAs (siRNAs) effectively reduced MLL2 mRNA expression (Fig. 1a). The efficiency of MLL2 siRNAs was verified by quantitative RT-PCR instead of immunoblotting, due to the technical difficulty in detecting MLL2 by immunoblotting; MLL2 is a large protein of ~600 kDa. We found that depletion of MLL2 resulted in a significant decrease in the level of H3K4 methylation during the period of serum starvation (Fig. 1b). Both the percentage of ciliated cells and the length of cilia were markedly increased by MLL2 depletion (Fig. 1c–e), indicating that MLL2 negatively regulates ciliary initiation and elongation. In addition, inhibition of MLL2 activity with the H3K4 methyltransferase inhibitor 5′-deoxy-5′-(methylthio)adenosine (MTA) promoted both the initiation and elongation of cilia (Fig. 1f–h). By contrast, the H3K9 methyltransferase inhibitor BIX-01294 did not obviously affect ciliary initiation or elongation (Supplementary Fig. S1).

We next analyzed the effects of the catalytic SET domain of MLL2 on ciliogenesis; we used the SET domain instead of full-length MLL2, due to unsuccessful expression of the full-length protein. We found that overexpression of GFP-MLL2-SET in RPE-1 cells decreased both the percentage of ciliated cells and the length of cilia compared with overexpression of green fluorescent protein (GFP) alone (Fig. 1i–k). Furthermore, MLL2 depletion-induced increase in ciliogenesis was partially rescued by GFP-MLL2-SET overexpression (Fig. 1i–k). Flow cytometric analysis revealed that depletion of MLL2 or overexpression of GFP-MLL2-SET did not significantly alter the cell cycle of RPE-1 cells (Supplementary Fig. S2), suggesting that the effect of MLL2 on ciliogenesis was independent of the cell cycle. Taken together, these results suggest that MLL2 plays a negative role in ciliogenesis through its H3K4 methyltransferase activity.

### MLL2 depletion downregulates actin-associated proteins to promote ciliogenesis

**Fig. 2:**
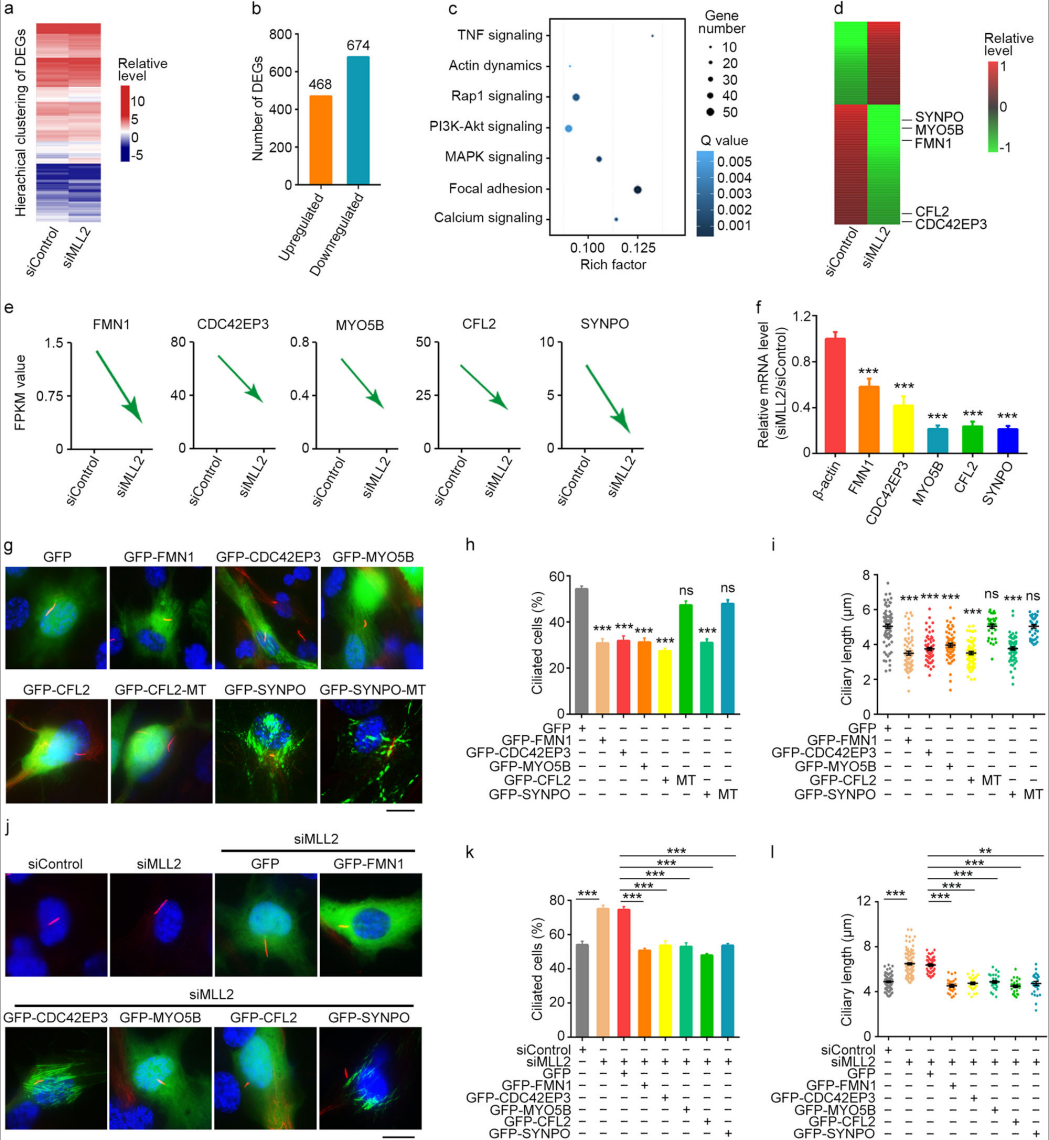
Downregulation of actin-associated proteins by MLL2 depletion underlies its effect on ciliogenesis. **a**, **b** RPE-1 cells were transfected with control or MLL2 siRNAs and serum-starved for 48 h. The hierarchical clustering of DEGs (**a**) and number of DEGs (**b**) were then obtained. **c** KEGG pathway analysis showing signaling pathways affected by MLL2 depletion. MAPK, mitogen-activated protein kinase; PI3K, phosphoinositide 3-kinase; TNF, tumor necrosis factor. **d** Heatmap showing the differential expression of proteins by MLL2 depletion. **e** FPKM value analysis of the relative expression of five actin-associated proteins in RPE-1 cells transfected with control or MLL2 siRNAs and serum-starved for 48 h. **f** Quantitative RT-PCR analysis of the relative mRNA levels of the indicated proteins upon MLL2 depletion. **g**–**i** Immunofluorescence images (**g**), percentage of ciliated cells (**h**, *n* = 100), and ciliary length (**i**, *n* = 30) for RPE-1 cells transfected with the indicated plasmids, serum-starved for 48 h, and stained with acetylated α-tubulin antibodies (red) and DAPI (blue). In the DDI/EEV mutant of CFL2 (CFL2-MT), Asp141, Asp142, and Ile143 were replaced by Glu, Glu, and Val, respectively. In the Δ752–903 mutant of SYNPO (SYNPO-MT), amino acids from 752 to 903 were deleted. Scale bar, 10 µm. **j**–**l** Immunofluorescence images (**j**), percentage of ciliated cells (**k**, *n* = 100), and ciliary length (**l**, *n* = 30) for RPE-1 cells transfected with the indicated siRNAs and/or plasmids, serum-starved for 48 h, and stained with acetylated α-tubulin antibodies (red) and DAPI (blue). Scale bar, 10 µm. **P < 0.01; ***P < 0.001. Error bars indicate SEM

To investigate whether MLL2 suppresses ciliogenesis through its role in transcriptional regulation, we performed RNA-sequencing (RNA-seq) to examine changes in transcription upon MLL2 depletion under serum-starvation conditions. Hierarchical clustering of differentially expressed genes (DEGs), which were defined as those exhibiting twofold upregulation or downregulation, revealed substantial difference upon MLL2 depletion; there were 468 genes upregulated and 674 genes downregulated in serum-starved MLL2-knockdown cells (Fig. 2a, b and Supplementary Table S1).

We then performed Kyoto Encyclopedia of Genes and Genomes (KEGG) pathway analysis for the RNA-seq data. Several signaling pathways were found to be significantly affected by MLL2 depletion under serum-starvation conditions, including the tumor necrosis factor, mitogen-activated protein kinase, and phosphoinositide 3-kinase-Akt signaling pathways (Fig. 2c). In addition, KEGG pathway analysis of the RNA-seq data revealed that MLL2 depletion affected five actin-associated proteins, including formin 1 (FMN1), cell division cycle 42 effector protein 3 (CDC42EP3), myosin 5B (MYO5B), cofilin 2 (CFL2), and synaptopodin (SYNPO), with significantly decreased FPKM (fragments per kilobase of transcript per million fragments mapped) values (Fig. 2c–e). Quantitative RT-PCR analysis further confirmed the downregulation of these five proteins in MLL2-depleted cells (Fig. 2f). This finding, together with the known linkage of actin dynamics to ciliary regulation, prompted us to investigate how MLL2 regulates actin dynamics.

We asked whether the regulation of actin-associated proteins by MLL2 mediates its suppression of ciliogenesis. Overexpression of GFP-FMN1, GFP-CDC42EP3, GFP-MYO5B, GFP-CFL2, or GFP-SYNPO significantly decreased both the percentage of ciliated cells and the length of cilia (Fig. 2g–i). By contrast, overexpression of the DDI/EEV mutant of CFL2 and the Δ752–903 mutant of SYNPO, which lose the actin-binding capacity^30,31^, were unable to inhibit ciliogenesis (Fig. 2g–i). We also found that siRNA-mediated knockdown of FMN1 or CFL2 could increase ciliogenesis (Supplementary Fig. S3). In addition, overexpression of GFP-FMN1, GFP-CDC42EP3, GFP-MYO5B, GFP-CFL2, or GFP-SYNPO could partially rescue MLL2 depletion-induced changes in ciliogenesis (Fig. 2j–l). These results suggest that regulation of actin-associated proteins contributes to the inhibitory role of MLL2 in ciliogenesis.

### MLL2 regulates actin dynamics

**Fig. 3:**
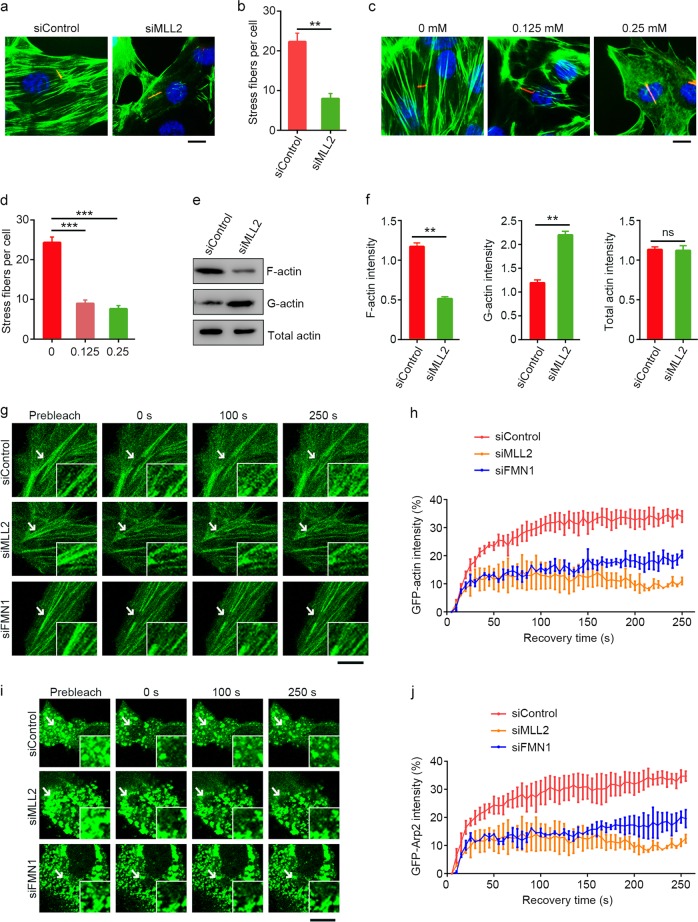
MLL2 regulates actin dynamics. **a**–**d** Immunofluorescence images (**a**, **c**) and quantification of stress fibers (**b**, **d**, *n* = 50) for RPE-1 cells transfected with control or MLL2 siRNAs (**a**, **b**) or treated with the indicated concentrations of MTA (**c**, **d**), serum-starved for 48 h, and stained with acetylated α-tubulin antibodies (red), FITC-phalloidin (green), and DAPI (blue). Scale bars, 5 µm. **e**, **f** Immunoblotting (**e**) and quantification (**f**) of F-actin, G-actin, and total actin from RPE-1 cells transfected with control or MLL2 siRNAs and serum-starved for 48 h. **g**, **h** RPE-1 cells were transfected with GFP-actin and control, MLL2, or FMN1 siRNAs and serum-starved for 48 h. Images were recorded at 5 s intervals following photobleaching of the indicated area (**g**) and fluorescence recovery at different time points was quantified (**h**, *n* = 10). Insets show higher magnifications of the bleaching regions. Scale bar, 10 µm. **i**, **j** RPE-1 cells were transfected with GFP-Arp2 and control, MLL2, or FMN1 siRNAs and serum-starved for 48 h. Images were recorded at 5 s intervals following photobleaching of the indicated area (**i**) and fluorescence recovery at different time points was quantified (**j**, *n* = 10). Insets show higher magnifications of the bleaching regions. Scale bar, 10 µm. **P < 0.01; ***P < 0.001; ns, not significant. Error bars indicate SEM

F-actin forms dynamic networks mainly comprising stress fibers and branched F-actin in the cell. Branched F-actin nucleated by the Arp2/3 complex is important for the formation of lamellipodia^32,33^ but inhibits ciliogenesis^19–23^. The regulation of actin-associated proteins by MLL2 suggests that it may affect actin dynamics. To test this, we examined the effect of MLL2 depletion on the morphology of F-actin networks in serum-starved RPE-1 cells. Control cells exhibited highly organized stress fibers, whereas these structures were reduced and disorganized in MLL2-depleted cells (Fig. 3a, b). Similar effects were observed in RPE-1 cells treated with the H3K4 methyltransferase inhibitor MTA (Fig. 3c, d).

Maintenance of proper actin dynamics in cells requires an exquisite regulation of the concentrations of globular-actin (G-actin) and F-actin by multiple actin-associated proteins. Thus, we examined the effect of MLL2 depletion on the distribution of F-actin and G-actin in serum-starved cells. We found that MLL2 depletion did not significantly affect total actin, but caused a dramatic decrease in F-actin and increase in G-actin (Fig. 3e, f). By fluorescence recovery after photobleaching (FRAP) experiments, we further found that depletion of MLL2 or FMN1 under serum-starvation conditions dramatically impaired the turnover of GFP-actin (Fig. 3g, h). In addition, FRAP experiments revealed that the turnover of GFP-Arp2 was inhibited in MLL2- or FMN1-depleted cells (Fig. 3i, j). These findings indicate that MLL2 plays a critical role in the regulation of actin dynamics.

### MLL2 regulates actin-mediated cellular processes

**Fig. 4:**
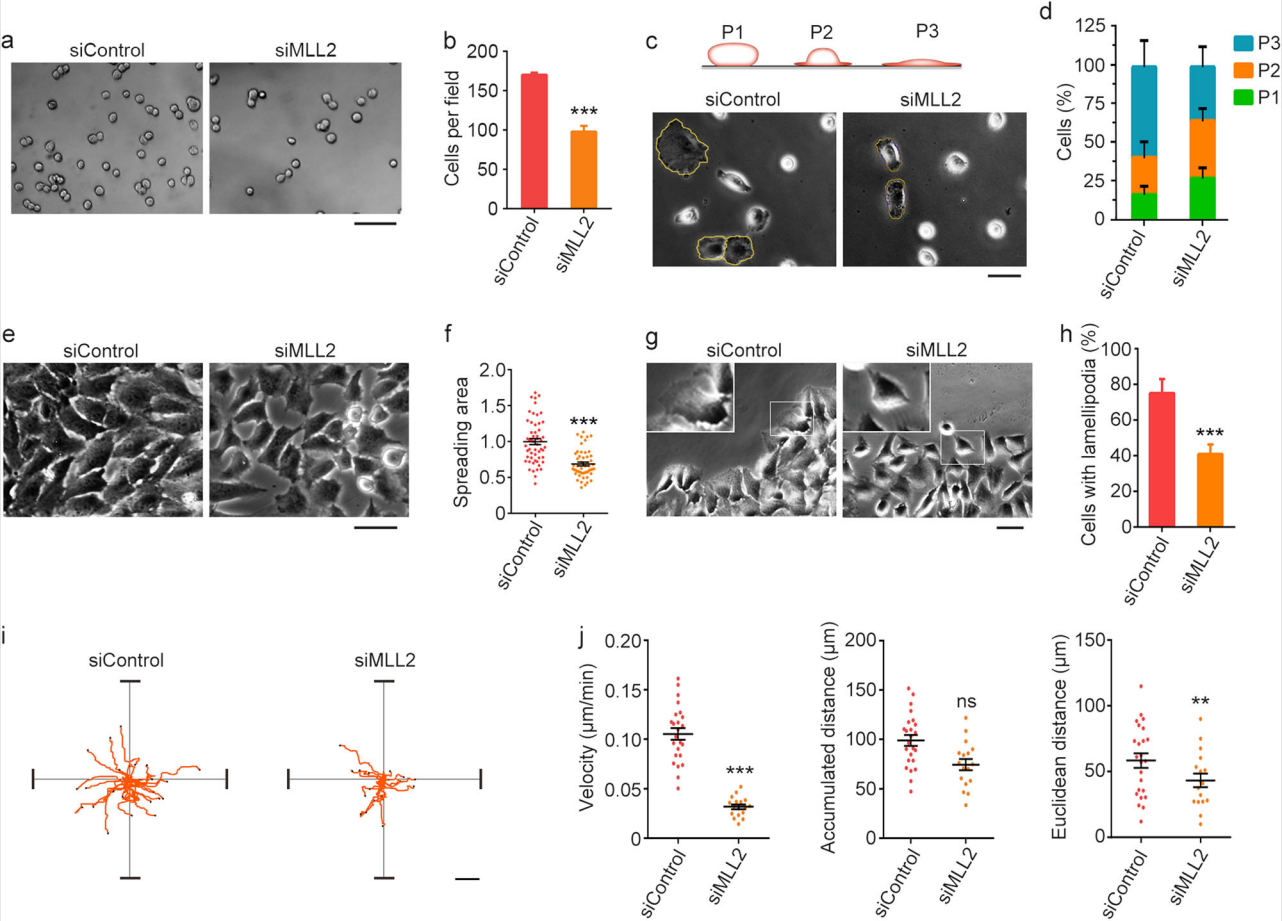
MLL2 regulates actin-mediated cellular processes. **a**, **b** RPE-1 cells were transfected with control or MLL2 siRNAs and plated on Matrigel-coated plates, and non-adherent cells were washed away after 30 min. Phase-contrast images were taken (**a**) and the number of adherent cells was quantified (**b**, *n* = 30). Scale bar, 50 µm. **c**, **d** RPE-1 cells were transfected with control or MLL2 siRNAs and plated on plastic plates after suspension. Phase-contrast images were taken (**c**) and the percentages of cells with different spreading stages were quantified (**d**, *n* = 200). Scale bar, 25 µm. **e**, **f** Phase-contrast images (**e**) and quantification of the spreading area (**f**, *n* = 50) for RPE-1 cells transfected with control or MLL2 siRNAs and serum-starved for 48 h. Scale bar, 25 µm. **g**, **h** Phase-contrast images (**g**) and quantification of the proportion of cells with lamellipodia (**h**, *n* = 200) for RPE-1 cells transfected with control or MLL2 siRNAs and scratched to induce migration. Insets show higher magnifications of the selected regions. **i**, **j** Migration trajectories (**i**) and various parameters of migration (**j**, *n* = 24 for siControl; *n* = 17 for siMLL2) for RPE-1 cells transfected with control or MLL2 siRNAs. Scale bar, 25 µm. **P < 0.01; ***P < 0.001; ns, not significant. Error bars indicate SEM

We then sought to investigate the effects of MLL2 on actin-mediated cellular processes, such as cell adhesion, spreading, and migration. We found that depletion of MLL2 significantly impaired the adhesion of RPE-1 cells (Fig. 4a, b). In addition, a greater proportion of MLL2-depleted cells remained rounded up compared with control cells (Fig. 4c, d), indicating that MLL2 knockdown interferes with cell spreading. This was confirmed by quantification of the spreading area; compared with control cells, MLL2-knockdown cells spread over a smaller area (Fig. 4e, f).

We noticed that MLL2-depleted cells formed rounded clusters with a paucity of lamellipodia (Fig. 4c, d). Scratch wound-healing assays also revealed that MLL2 depletion dramatically abolished lamellipodium formation at the leading edge (Fig. 4g, h). These data suggest that depletion of MLL2 in RPE-1 cells might affect their capacity to migrate. Consistent with this notion, analysis of the migration trajectories of RPE-1 cells revealed that knockdown of MLL2 reduced both the velocity of cell migration and the distance traveled from the origin (Fig. 4i, j). Collectively, these findings demonstrate that MLL2 is a critical regulator of actin-mediated cellular processes.

### MLL2 depletion promotes ciliogenesis by disrupting branched F-actin

**Fig. 5:**
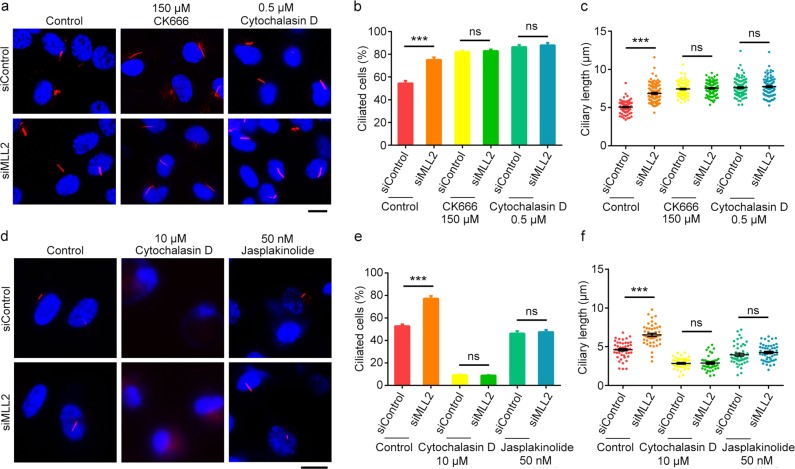
MLL2 depletion promotes ciliogenesis by disrupting branched F-actin. **a**–**c** Immunofluorescence images (**a**), percentage of ciliated cells (**b**, *n* = 200), and ciliary length (**c**, *n* = 100) for RPE-1 cells transfected with control or MLL2 siRNAs, serum-starved for 48 h, and treated with CK666 (150 µM) or cytochalasin D (0.5 µM) for 4 h. Cells were stained with acetylated α-tubulin antibodies (red) and DAPI (blue). Scale bar, 10 µm. **d**–**f** Immunofluorescence images (**d**), percentage of ciliated cells (**e**, *n* = 200), and ciliary length (**f**, *n* = 50) for RPE-1 cells transfected with control or MLL2 siRNAs, serum-starved for 48 h, and treated with cytochalasin D (10 µM) or jasplakinolide (50 nM) for 4 h. Cells were stained with acetylated α-tubulin antibodies (red) and DAPI (blue). Scale bar, 10 µm. ***P < 0.001; ns, not significant. Error bars indicate SEM

We next asked whether the alteration of actin dynamics is required for MLL2 to inhibit ciliogenesis. To answer this question, RPE-1 cells were transfected with control or MLL2 siRNAs, serum-starved, and treated with CK666, an Arp2/3-specific inhibitor that prevents the formation of branched F-actin. We found that CK666 increased ciliogenesis both in control cells and in MLL2-depleted cells; however, their difference in ciliogenesis was abolished in the presence of CK666 (Fig. 5a–c). Similarly, treatment of RPE-1 cells with low-dose cytochalasin D (0.5 µM), which predominantly affects branched F-actin, could increase ciliogenesis both in control cells and in MLL2-depleted cells (Fig. 5a–c). Low-dose cytochalasin D also abolished the difference in ciliogenesis between control and MLL2-depleted cells (Fig. 5a–c). These results suggest that disruption of branched F-actin mediates the action of MLL2 depletion to induce ciliogenesis.

To gain more insights into how actin dynamics impacts the effect of MLL2 on ciliogenesis, we treated cells with high-dose cytochalasin D (10 µM), which is known to cause complete depolymerization of F-actin in the cell. We found that high-dose cytochalasin D dramatically inhibited ciliogenesis both in control cells and in MLL2-depleted cells, and also abrogated the difference in ciliogenesis between control and MLL2-depleted cells (Fig. 5d–f). Furthermore, treatment with jasplakinolide, which causes abnormal stabilization of cellular F-actin, also inhibited ciliogenesis in control and MLL2-depleted cells, and abrogated their difference in ciliogenesis (Fig. 5d–f). Taken together, these data suggest that alteration of actin dynamics contributes to the effect of MLL2 on ciliogenesis.

### MLL2 depletion stimulates the transport of ciliary vesicles to the basal body

**Fig. 6:**
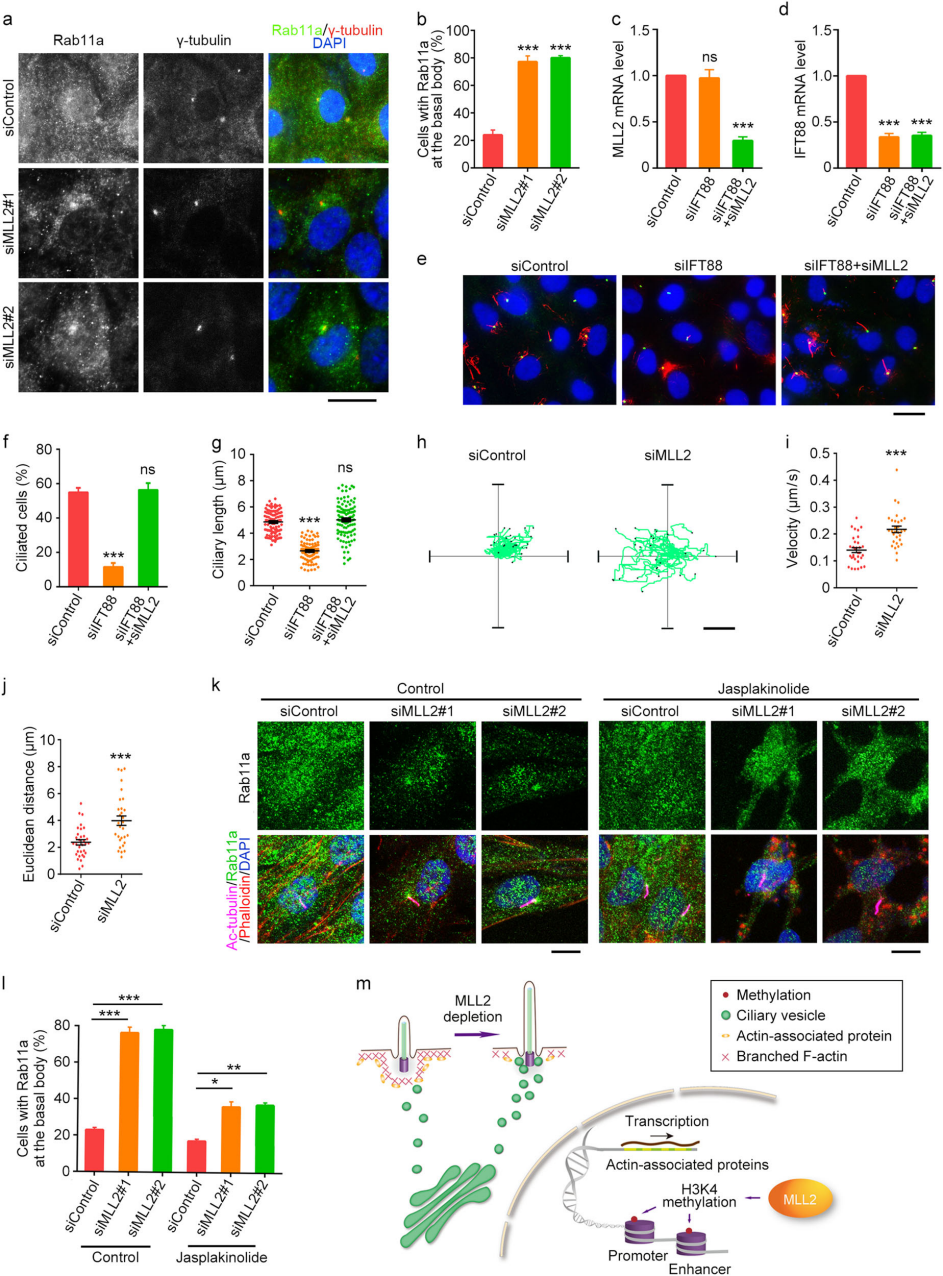
Knockdown of MLL2 expression stimulates the transport of ciliary vesicles to the basal body. **a**, **b** Immunofluorescence images (**a**) and percentage of cells with Rab11a at the basal body (**b**, *n* = 100) for RPE-1 cells transfected with control or MLL2 siRNAs, serum-starved for 24 h, and stained with antibodies against Rab11a (green) and γ-tubulin (red), and DAPI (blue). Scale bar, 10 µm. **c**, **d** Quantitative RT-PCR analysis of the relative mRNA levels of MLL2 (**c**) and IFT88 (**d**) for RPE-1 cells transfected with the indicated siRNAs and serum-starved for 48 h. **e**–**g** Immunofluorescence images (**e**), percentage of ciliated cells (**f**, *n* = 200), and ciliary length (**g**, *n* = 80) for RPE-1 cells transfected with the indicated siRNAs and serum-starved for 24 h. Cells were stained with antibodies against γ-tubulin (green) and acetylated α-tubulin (red), and DAPI (blue). Scale bar, 10 µm. **h**–**j** Movement trajectories (**h**), velocity (**i**), and Euclidean distance (**j**) of GFP-Rab11a vesicles in RPE-1 cells transfected with GFP-Rab11a and control or MLL2 siRNAs and serum-starved for 24 h (*n* = 32 for siControl; *n* = 29 for siMLL2). Scale bar, 4 µm. **k**, **l** Immunofluorescence images (**k**) and percentage of cells with Rab11a at the basal body (**l**, *n* = 100) for RPE-1 cells transfected with control or MLL2 siRNAs, serum-starved for 24 h, and treated with jasplakinolide (50 nM) for 24 h. Cells were stained with antibodies against Rab11a (green) and acetylated α-tubulin (red), and DAPI (blue). Scale bar, 5 µm. **m** Molecular model for the role of MLL2 in ciliary regulation. MLL2 depletion downregulates actin-associated proteins, disrupts branched F-actin, and stimulates the transport of ciliary vesicles to the basal body, thereby promoting ciliogenesis. *P < 0.05; **P < 0.01; ***P < 0.001; ns, not significant. Error bars indicate SEM

Inhibition of branched F-actin has been reported to induce ciliogenesis by accumulating Rab11a-positive vesicles around the basal body^34^. Consistent with this finding, we found that in serum-starved MLL2-knockdown RPE-1 cells, Rab11a-positive vesicles were more prevalent around the basal body compared with control cells (Fig. 6a, b). MLL2 knockdown did not significantly affect the level of total cellular Rab11a (Supplementary Fig. S4a). In addition, we found that overexpression of GFP-MLL2-SET could rescue the accumulation of Rab11a vesicles at the basal body induced by MLL2 depletion (Supplementary Fig. S4b, c).

IFT88 is known to transport molecules required for ciliary assembly and cells transfected with IFT88 siRNAs fail to grow cilia or produce normal-length cilia (Fig. 6e–g)^35^. Notably, knockdown of MLL2 could rescue the ciliary defects present in IFT88-depleted cells (Fig. 6c–g), suggesting that MLL2 knockdown facilitates ciliary recruitment of IFT particles through the endocytic recycling pathway. Consistent with this notion, we found that knockdown of MLL2 under serum-starvation conditions markedly increased the velocity and distance of GFP-Rab11a vesicle movement in RPE-1 cells (Fig. 6h–j). We next assessed the role of actin regulation in MLL2-dependent changes in ciliary vesicle transport. Stabilization of F-actin by jasplakinolide partially prevented the assembly of Rab11a vesicles in MLL2-depleted cells (Fig. 6k, l). These results support the notion that MLL2 depletion-induced disruption of branched F-actin stimulates vesicle trafficking to the basal body to promote ciliogenesis.

## Discussion

Primary cilia play important roles in coordinating heart development^4^ and defects in cilia are associated with CHD^3^. MLL2 gene mutations are responsible for the majority of cases of Kabuki syndrome, which is frequently associated with CHD^36^. MLL2 has been reported to control cardiac lineage differentiation by promoting H3K4 methylation at cardiac-specific genes^29,37^. In addition, MLL2 is required for the regulation of ion transport and cell cycle gene expression in cardiac precursors and cardiomyocytes during cardiogenesis^29^. Analysis of the high proportion of Kabuki syndrome patients with CHD revealed that MLL2-associated CHD had phenotypes similar to those observed in ciliary dysfunction-associated CHD, such as left-side heart anomalies^38^. However, the function of MLL2 alteration in CHD remains elusive. In this study, we report that MLL2 depletion results in ciliary abnormality, which is linked to the downregulation of actin-associated proteins, alteration of actin dynamics, and increase in vesicle transport to the basal body (Fig. 6m), providing novel insights into the role of MLL2 in the development of CHD.

Branched F-actin has been reported to impact ciliogenesis by modulating vesicle trafficking^19^. Suppression of branched F-actin serves as a way to modulate the function and dynamics of Rab11a-associated vesicles for ciliary formation. Our data demonstrate that depletion of MLL2 impairs actin dynamics and actin-mediated cellular processes, and that both the Arp2/3-specific inhibitor CK666 and the F-actin-destabilizing agent cytochalasin D at doses disrupting branched F-actin could abolish the difference in ciliogenesis between control and MLL2-depleted cells. These results support the notion that inhibiting branched F-actin is an important part of how MLL2 depletion promotes ciliogenesis. Our data also show that MLL2 depletion induces vesicle accumulation around the basal body, similar to the phenotype of cells with impaired branched F-actin, suggesting a role for the MLL2/branched F-actin axis in promoting ciliary vesicle trafficking.

The present study has identified five actin-associated proteins that are downregulated by MLL2 depletion under serum-starvation conditions, including FMN1, CDC42EP3, MYO5B, CFL2, and SYNPO. These five proteins and MLL2 were not identified in the previous genomic screen using RNA interference, which revealed 49 ciliogenesis regulators, including proteins involved in actin dynamics and vesicle transport^19^. Among the five actin-associated proteins identified in the present study, FMN1 is an actin-nucleating protein that promotes the generation of unbranched F-actin in vitro and functions in the formation of radial actin cables in the cell^39^. Intriguingly, our data show that alteration of FMN1 expression affects the turnover of Arp2, suggesting a potential crosstalk between unbranched and branched F-actin in the cell. CDC42EP3 is known to stimulate the formation of F-actin and pseudopodia^40^. CFL2 and other confilins control actin turnover in cells by severing and nucleation, depending on the local concentrations of active cofilins^41^. SYNPO regulates actin dynamics to mediate cell shaping and motility in highly specified cell compartments^42^. It remains to be determined how these proteins coordinate the assembly and disassembly of actin filaments in highly dynamic cellular processes, such as cell adhesion, lamellipodium formation, and ciliogenesis.

Members of the MYO5 family are actin-based motor proteins that play critical roles in vesicle transport. Recent studies have identified MYO5A as a ciliary component and revealed that this protein is required for ciliogenesis^11,17^. Furthermore, MYO5A has been shown to promote preciliary vesicle transport to the mother centriole during ciliogenesis^18^. In this study, our data demonstrate that MYO5B is a negative regulator of ciliogenesis, manifesting the complexity and specificity of MYO5 family proteins in ciliary regulation. It will be interesting to examine in the future how the MYO5B-actin axis is involved in the regulation of ciliogenesis by MLL2.

Understanding how actin polymerization affects heart development and function is an emerging and exciting area of research. The newly identified roles for MLL2 in the regulation of actin dynamics and ciliogenesis has important implications in health and disease. MLL2 appears to be capable of integrating epigenetic and cytoskeletal regulation in response to extracellular cues. It will be important for future investigations to determine whether MLL2-dependent changes in the expression of actin-associated proteins are required for its function in CHD. In addition, further studies are warranted to study whether MLL2 is involved in the regulation of actin dynamics and ciliogenesis during heart development.

## Materials and methods

### Materials

Mammalian expression plasmids for GFP-MLL2-SET, GFP-FMN1, GFP-CDC42EP3, GFP-MYO5B, GFP-CFL2, GFP-SYNPO, Arp2, β-actin, and Rab11a were constructed by insertion of the respective cDNAs into the pEGFP-C1 vector. The DDI/EEV mutant of CFL2 and the Δ752–903 mutant of SYNPO were generated by site-directed mutagenesis and PCR. MLL2 siRNAs (#1: 5′-GCAUGAAGCCGCAGCAAUU-3′; #2: 5′-GUGCCAAGUGCAUGUUCUU-3′), FMN1 siRNAs (#1: 5′-GCAGAAACCUGUCUCCAAA-3′; #2: 5′-GCAAGACAACCAGAAGUAA-3′), and CFL2 siRNAs (#1: 5′-GUUCGACACUUGGAGAGAA-3′; #2: 5′-GGGAGGCAAUGUAGUAGUU-3′) were synthesized by Ribobio. Antibodies against α-tubulin, acetylated α-tubulin, H3K4me1 (Abcam), β-actin (Sigma-Aldrich), γ-tubulin (Sigma-Aldrich), and Rab11a (Invitrogen) were purchased from the indicated sources. Horseradish peroxidase-conjugated secondary antibodies were from Santa Cruz Biotechnology. Fluorescein isothiocyanate (FITC)- and rhodamine-conjugated secondary antibodies were from Jackson ImmunoResearch Laboratories. FITC-conjugated phalloidin was from Sigma-Aldrich and 4′,6-diamidino-2-phenylindole (DAPI) was purchased from Songon Biotech. MTA, BIX-01294, CK666 (Sigma-Aldrich), cytochalasin D (EMD Millipore), and jasplakinolide (BioVision) were from the indicated sources.

### Cell culture and transfection

RPE-1 cells were obtained from the American Type Culture Collection and cultured in the Dulbecco’s modified Eagle’s medium/F12 medium supplemented with 10% fetal bovine serum. Plasmids and siRNAs were transfected with Lipofectamine 3000 and Lipofectamine RNAimax (Thermo Fisher Scientific), respectively.

### Fluorescence microscopy

Cells grown on glass coverslips were fixed with 4% paraformaldehyde in phosphate-buffered saline (PBS) for 25 min, followed by permeabilization in 0.5% Triton X-100 in PBS for 8 min, or fixed with methanol at −20 °C for 3 min. Cells were blocked with 2% bovine serine albumin in PBS and incubated with primary antibodies and then FITC- or rhodamine-conjugated secondary antibodies, followed by staining with DAPI as described previously^43^. For visualization of actin filaments, cells were stained with FITC-conjugated phalloidin for 30 min. Coverslips were mounted and examined with an Axio Imager Z1 fluorescence microscope (Carl Zeiss).

### FRAP experiments

Cells transfected with GFP-actin or GFP-Arp2 were trypsinized and replated on confocal dishes and examined with an LSM710 confocal microscope (Carl Zeiss). Images were recorded at 5 s intervals after photobleaching of the selected area to 30%. Fluorescence recovery at different time points was then quantified.

### Live-cell imaging of cell migration and vesicle movement

Cells were examined with an Axio Observer Z1 fluorescence microscope (Carl Zeiss). Time-lapse images were acquired at 5 min intervals for 5 h (for cell migration) or 10 s intervals for 10 min (for vesicle movement). Cell migration and vesicle movement trajectories were obtained by using the ImageJ software with the manual-tracking and chemotaxis-migration tool plugins. The velocity of cell migration or vesicle movement, the Euclidean distance, and the accumulated distance were then calculated.

### Cell adhesion and spreading

For cell adhesion, actively growing cells were trypsinized and replated on Matrigel-coated plates. Non-adherent cells were washed away with PBS 30 min later and the remaining cells were examined. For cell spreading, cells were replated on uncoated plastic plates and examined 2 h later. To examine lamellipodia, cells cultured in 24-well plates were scratched with a 20 μL pipette tip to generate the wound. Cells were then washed twice with PBS to remove the cell debris. Photographs of the wound were taken 3 h later to detect lamellipodia.

### Flow cytometry

RPE-1 cells were trypsinized and washed twice with ice-cold PBS. Then cells were fixed with 70% ethanol and incubated with propidium iodide/RNase A solution for 30 min. Samples were analyzed with a BD FACS Calibur flow cytometer (BD Biosciences).

### Immunoblotting

Proteins were resolved by SDS-polyacrylamide gel electrophoresis (PAGE) and transferred onto polyvinylidene difluoride membranes (Millipore). The membranes were blocked in Tris-buffered saline containing 0.1% Tween 20 and 5% fat-free milk for 2 h at room temperature, and incubated first with primary antibodies and then with horseradish peroxidase-conjugated secondary antibodies as described previously^44^. Specific proteins were visualized with enhanced chemiluminescence detection reagent (Pierce).

### Isolation of G-actin and F-actin

The G-actin and F-actin fractions were prepared using the G-actin/F-actin In Vivo Assay Kit (Cytoskeleton). In brief, cells were homogenized in an F-actin-stabilizing lysis buffer at 37 °C for 10 min. The homogenate was centrifuged at 1000 × *g* at room temperature for 5 min to remove nuclei, unbroken cells, and cell debris. The lysate was centrifuged at 100,000 × *g* at 37 °C for 1 h. The supernatant (G-actin fraction) was then collected gently and the pellet (F-actin fraction) was incubated in an F-actin-depolymerizing buffer on ice. SDS-PAGE and immunoblotting were then performed to quantify the distribution of different actin fractions.

### Quantitative RT-PCR analysis

A total of 2 µg of total RNA was used for each reverse-transcription reaction using the Superscript III First Strand Synthesis System with oligo-dT primers (Invitrogen). Quantitative real-time PCR was then performed using an Applied Biosystems 7500 HT Sequence Detection System with the Power SYBR Green PCR Master Mix Kit (Applied Biosystems). All of the reactions were performed in triplicate with β-actin as the control.

### RNA-seq and data analysis

Total RNA was isolated using the TRIzol reagent (Invitrogen), according to the manufacturer’s protocol. The mRNAs were enriched by oligo(dT) beads, fragmented, and reverse transcribed into cDNAs with random primers. The cDNA fragments were then purified, PCR amplified, and sequenced with the BGISEQ-500 sequencer. The expressed values of each sample were calculated by Cufflinks and Cuffdiff based on the FPKM function. The genes with FPKM values changed over twofold were defined as significantly changed genes. The heatmap chart was drawn based on the FPKM values using the gplots heatmap.2 function of the R program. The hierarchical clustering of DEGs was analyzed based on the differential gene pairs between groups. Gene enrichment was conducted by KEGG pathway analysis.

### Statistics

Analysis of statistical significance was performed by the Student’s *t*-test.

## Supplementary information

Supplementary information.

## Supplementary information

**Supplementary Information** accompanies the paper at (10.1038/s41421-019-0100-3).
