# Supplementary material for: Mixed-lineage leukemia protein 2 suppresses ciliary assembly by the modulation of actin dynamics and vesicle transport
Source: Cell Discov. 2019 Jun 25;5:33. doi: 10.1038/s41421-019-0100-3 (PMC6591415; doi:10.1038/s41421-019-0100-3)
Supplement: Supplementary file 1 — Supplementary information. [file 41421_2019_100_MOESM1_ESM.pdf]

Supplementary information

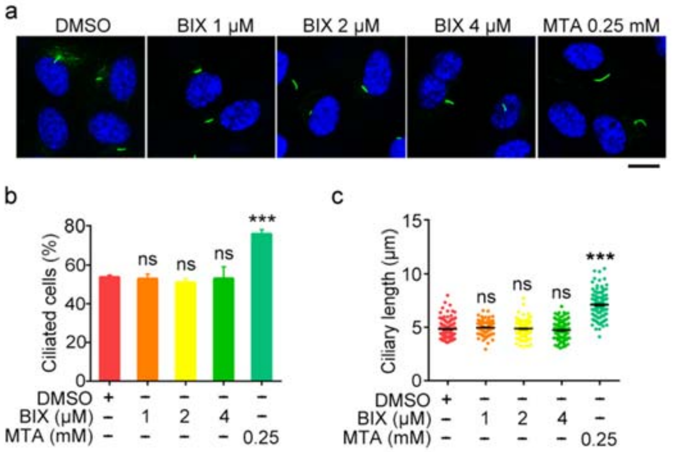

Fig. S1 The H3K9 methyltransferase inhibitor BIX-01294 does not affect ciliogenesis. Immunofluorescence images (**a**), percentage of ciliated cells (**b**, n = 200), and ciliary length (**c**, n = 100) for RPE-1 cells treated with the indicated concentrations of BIX-01294 (BIX) or MTA, serum-starved for 48 hours, and stained with acetylated α-tubulin antibodies (green) and DAPI (blue). Scale bar, 10 μm. ****P*< 0.001; ns, not significant. Error bars indicate SEM.

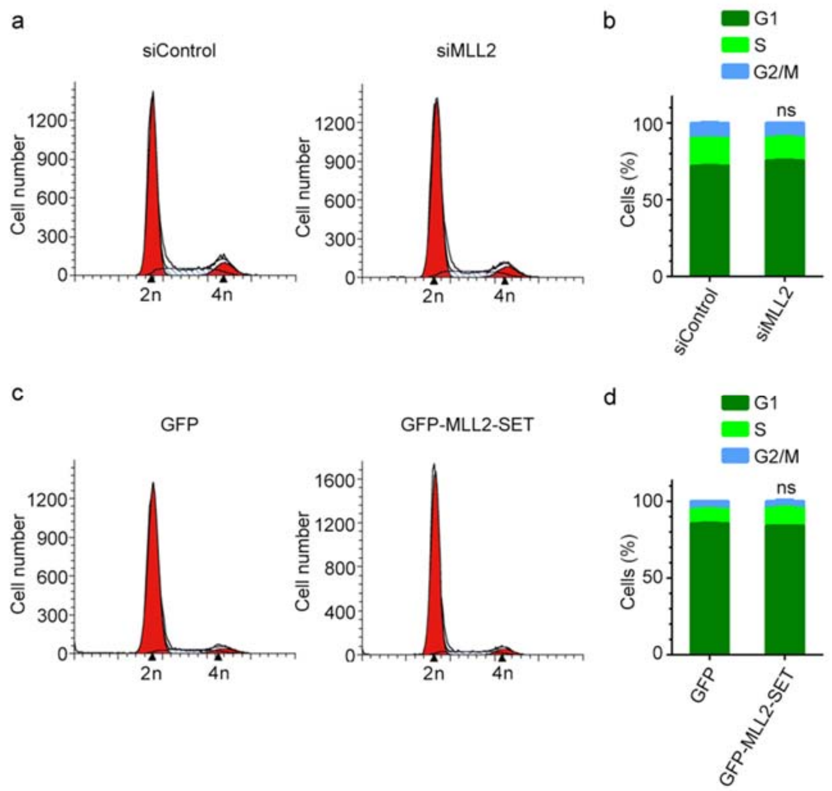

Fig. S2 Depletion of MLL2 or overexpression of its SET domain does not alter the cell cycle. a, **b** RPE-1 cells were transfected with control or MLL2 siRNAs for 48 hours, and the cell cycle was analyzed by flow cytometry (**a**). The percentages of cells in G1, S, and G2/M phases were then quantified (**b**). **c**, **d** RPE-1 cells were transfected with GFP or GFP-MLL2-SET for 48 hours, and the cell cycle was analyzed by flow cytometry (**c**). The percentages of cells in G1, S, and G2/M phases were then quantified (**d**). 2n, cells in G1; 4n, cells in G2/M. ns, not significant. Error bars indicate SEM.

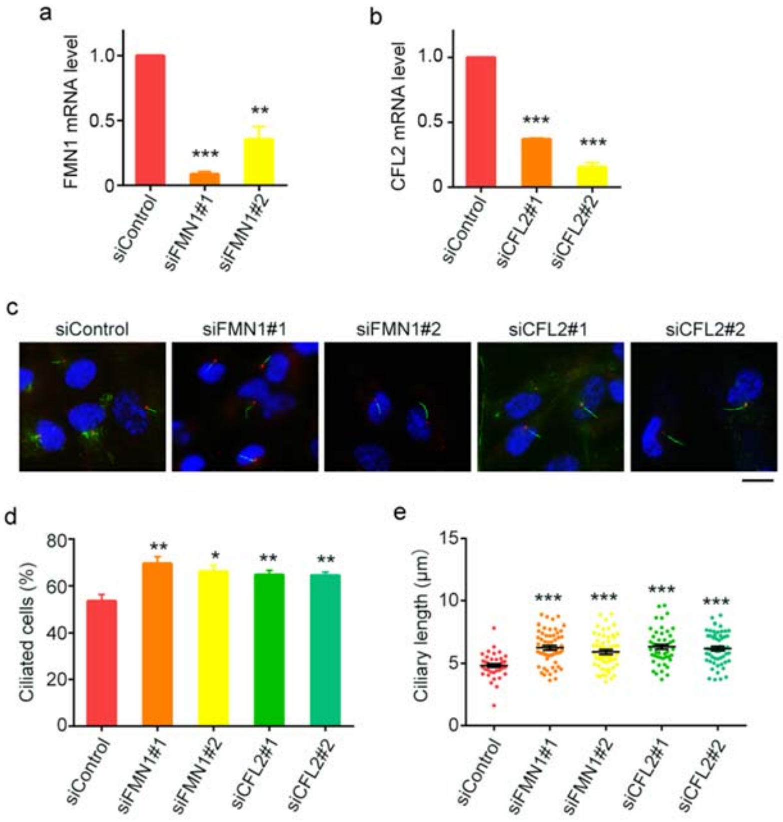

Fig. S3 Knockdown of FMN1 or CFL2 promotes ciliogenesis. **a**, **b** Quantitative RT-PCR analysis of FMN1 and CFL2 mRNA expression in RPE-1 cells transfected with the indicated siRNAs and serum-starved for 48 hours. **c-e** Immunofluorescence images (**c**), percentage of ciliated cells (**d**, n = 200), and ciliary length (**e**, n = 50) for RPE-1 cells transfected with the indicated siRNAs, serum-starved for 48 hours, and stained with antibodies against γ-tubulin (red) and acetylated α-tubulin (green), and DAPI (blue). Scale bar, 10 μm. *P< 0.05; **P< 0.01; ***P< 0.001. Error bars indicate SEM.

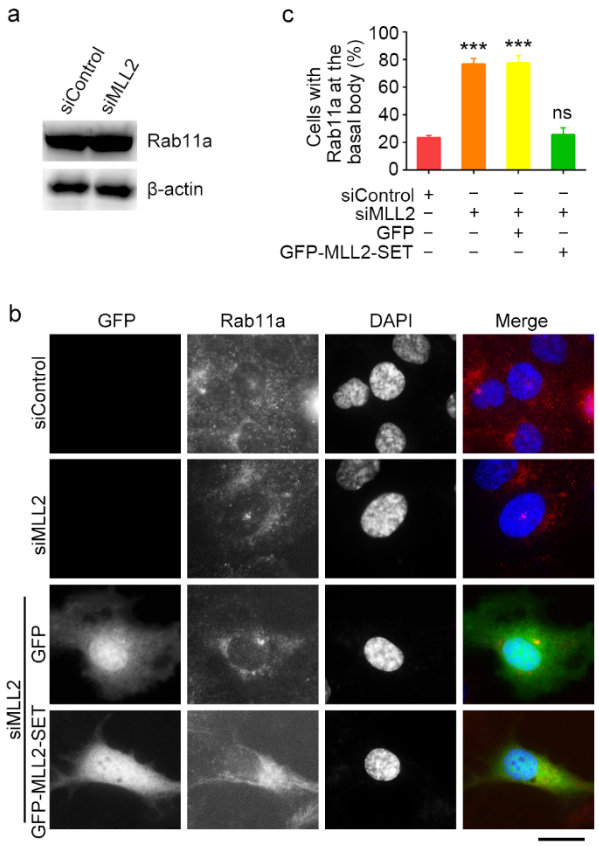

Fig. S4 Overexpression of GFP-MLL2-SET could rescue MLL2 depletion-induced accumulation of Rab11a vesicles at the basal body. a Immunoblot analysis of Rab11a and β-actin in RPE-1 cells transfected with control or MLL2 siRNAs and serum-starved for 48 hours. **b**, **c** Immunofluorescence images (**b**) and percentage of cells with Rab11a at the basal body (**c**, n = 100) for RPE-1 cells transfected with the indicated siRNAs and plasmids, serum-starved for 24 hours, and stained with Rab11a antibodies (red) and DAPI (blue). Scale bar, 10 µm. ****P*< 0.001; ns, not significant. Error bars indicate SEM.

Table S1. Differentially expressed genes by MLL2 depletion under serum-starvation conditions.

ID	Symbol	Length	FPKM-siControl	FPKM-siMLL2	siMLL2/ siControl
642799	NPIPA2	1088	0.01	13.41	Upregulation
100996928	C7orf55-LUC7L2	2747	0.01	11.68	Upregulation
4312	MMP1	2081	0.01	5	Upregulation
387522	TMEM189-UBE2V1	2925	0.01	4.84	Upregulation
8293	SERF1A	1935	0.01	4.45	Upregulation
11272	PRR4	585	0.01	3.3	Upregulation
3485	IGFBP2	1200.34	0.01	2.93	Upregulation
101180901	IPO11-LRRC70	1818	0.01	2.47	Upregulation
100528030	POC1B-GALNT4	4740	0.01	2.29	Upregulation
29907	SNX15	1957	0.01	2.24	Upregulation
100533105	C8orf44-SGK3	4563	0.01	2.04	Upregulation
100533955	SENP3-EIF4A1	4303	0.01	1.57	Upregulation
401428	OR2A20P	930	0.01	1.51	Upregulation
100526760	ABHD14A-ACY1	1879	0.01	1.46	Upregulation
100529855	ZNF625-ZNF20	3418	0.01	1.24	Upregulation
414224	AGAP12P	2141	0.01	1.07	Upregulation
378108	TRIM74	1341	0.01	1.03	Upregulation
135	ADORA2A	2110.34	0.08	5.81	Upregulation
9884	LRRC37A	5177	0.01	0.64	Upregulation
3381	IBSP	1595	0.46	28.11	Upregulation
283767	GOLGA6L1	3822	0.01	0.6	Upregulation
100631383	FAM47E-STBD1	3060	0.01	0.53	Upregulation
729540	RGPD6	7640	0.01	0.5	Upregulation
2615	LRRC32	4222	0.02	0.99	Upregulation
5744	PTHLH	1473.62	0.57	25.45	Upregulation
2769	GNA15	2311	0.05	2.04	Upregulation
11009	IL24	1958.98	2.08	79.43	Upregulation
374	AREG	1290	1.32	43.11	Upregulation
5743	PTGS2	4507	26.17	811.17	Upregulation
100302650	BRE-AS1	1667	0.07	1.74	Upregulation
1440	CSF3	1598.45	1.86	43.39	Upregulation
1960	EGR3	4342	0.05	1.1	Upregulation
64388	GREM2	4199	0.4	8.56	Upregulation
5617	PRL	1147.36	0.1	2.05	Upregulation
5168	ENPP2	2370.19	0.23	4.52	Upregulation
102724594	U2AF1L5	989.82	0.37	5.74	Upregulation
7130	TNFAIP6	1439	43.74	662.71	Upregulation
1959	EGR2	2836.15	0.14	1.96	Upregulation
387763	C11orf96	1361	1.09	15.21	Upregulation
4681	NBL1	1997.85	3.62	49.93	Upregulation
1880	GPR183	1801	1.23	16.75	Upregulation
1958	EGR1	3136	6.31	84.07	Upregulation
2262	GPC5	2966	0.21	2.75	Upregulation

2560	GABRB1	2226	0.09	1.09	Upregulation
3777	KCNK3	3978	0.86	10.2	Upregulation
9214	FCMR	2773	2.15	25.03	Upregulation
283208	P4HA3	2299	0.6	6.8	Upregulation
3589	IL11	2346.94	145.09	1531.65	Upregulation
1281	COL3A1	5490	0.4	4.19	Upregulation
9066	SYT7	6188	0.1	1.04	Upregulation
56936	CCDC177	4179	0.17	1.75	Upregulation
100861555	LINC00565	2494	0.08	0.8	Upregulation
5138	PDE2A	4146	0.05	0.5	Upregulation
5270	SERPINE2	2233.92	183.57	1775.76	Upregulation
2893	GRIA4	4321.33	0.06	0.55	Upregulation
8738	CRADD	1204.93	2.83	24.4	Upregulation
392232	LOC392232	2626	0.28	2.4	Upregulation
3164	NR4A1	2706.12	5.65	48.03	Upregulation
374946	DRAXIN	1773	0.48	4.05	Upregulation
597	BCL2A1	899.98	6.62	55.25	Upregulation
101929319	LOC101929319	1880	0.25	2.06	Upregulation
613037	LOC613037	3455	0.92	7.56	Upregulation
5798	PTPRN	3603.77	0.67	5.36	Upregulation
100137049	PLA2G4B	2752	1.34	10.58	Upregulation
650	BMP2	3191	8.49	65.11	Upregulation
3579	CXCR2	2672	1.78	13.59	Upregulation
2710	GK	4560.23	1.64	12.47	Upregulation
8013	NR4A3	5635	1.2	8.62	Upregulation
7056	THBD	4048	1.06	7.6	Upregulation
2827	GPR3	2149	0.64	4.01	Upregulation
2353	FOS	2158	0.83	5.16	Upregulation
1116	CHI3L1	1867	2.66	16.37	Upregulation
3484	IGFBP1	1660	40.78	246.54	Upregulation
2494	NR5A2	4922.99	0.13	0.76	Upregulation
55350	VNN3	1736.18	1.35	7.84	Upregulation
1634	DCN	4375.77	4.34	25.17	Upregulation
92162	TMEM88	807.78	0.73	4.15	Upregulation
57556	SEMA6A	6950	0.06	0.34	Upregulation
8942	KYNU	1688.59	12.18	67.23	Upregulation
6909	TBX2	3396	5.42	29.86	Upregulation
439965	FAM35DP	3294	0.24	1.3	Upregulation
10736	SIX2	2178	1.98	10.52	Upregulation
727751	LOC727751	3054.73	0.58	3.07	Upregulation
2354	FOSB	3766.03	0.61	3.16	Upregulation
441024	MTHFD2L	2372	3.59	18.49	Upregulation
57761	TRIB3	2294.49	4.99	25.44	Upregulation
58489	ABHD17C	2408	11.39	56.94	Upregulation
9536	PTGES	1787	38.25	182.84	Upregulation
147744	TMEM190	593	1.67	7.98	Upregulation
28231	SLCO4A1	2796	1.31	6.25	Upregulation
100529207	RAD51L3-RFFL	3904	0.33	1.56	Upregulation

1645	AKR1C1	1384	1.1	5.1	Upregulation
6364	CCL20	849.77	15.94	72.03	Upregulation
23753	SDF2L1	876	4.19	18.83	Upregulation
541472	LOC541472	1374	0.7	3.09	Upregulation
3569	IL6	1187.75	1015.77	4474.16	Upregulation
5452	POU2F2	6261.58	2.91	12.71	Upregulation
6348	CCL3	813	1.47	6.39	Upregulation
2921	CXCL3	1166	63.05	274.01	Upregulation
1909	EDNRA	3753.46	6.76	29.3	Upregulation
388	RHOB	2387	55.02	238.11	Upregulation
8870	IER3	1254	215.91	928.46	Upregulation
8325	FZD8	3186	3.09	13.25	Upregulation
90226	UCN2	1546	4.32	18.34	Upregulation
284021	MILR1	1432.41	5.71	24.02	Upregulation
3976	LIF	3980.93	39.04	164.07	Upregulation
3486	IGFBP3	2620	176.23	737.68	Upregulation
7975	MAFK	3350	21.42	88.52	Upregulation
622	BDH1	3412.32	3.08	12.69	Upregulation
2919	CXCL1	1184.05	192.92	792.35	Upregulation
4953	ODC1	2060.59	62.32	255.32	Upregulation
5343	PLGLB1	3011	0.43	1.75	Upregulation
2192	FBLN1	2581.86	0.38	1.54	Upregulation
26207	PITPNC1	6374.16	3.21	12.94	Upregulation
84557	MAP1LC3A	1048	2.25	8.89	Upregulation
202915	TMEM184A	6293	5.67	22.02	Upregulation
22999	RIMS1	5006.32	0.28	1.08	Upregulation
10135	NAMPT	4593	161.31	613.53	Upregulation
100132062	LOC100132062	4370	1.71	6.42	Upregulation
6775	STAT4	2793.95	3.18	11.91	Upregulation
7980	TFPI2	2443.64	97.47	362.73	Upregulation
100534612	C1QTNF3-AMACR	3612	0.25	0.93	Upregulation
101929780	NBPF25P	3827	1.91	7.09	Upregulation
101059953	NPIPA8	1165	8.54	31.55	Upregulation
23193	GANAB	3925.59	53.25	191.73	Upregulation
100128071	FAM229A	716	1.52	5.47	Upregulation
51129	ANGPTL4	1897.6	23	81.99	Upregulation
103625681	LLPH-AS1	623	1.96	6.98	Upregulation
50486	G0S2	978	496.66	1760.62	Upregulation
2920	CXCL2	1234	56.56	200.32	Upregulation
171483	FAM9B	2103	0.57	2	Upregulation
56892	C8orf4	1841	0.75	2.63	Upregulation
6525	SMTN	3329.24	13.26	46.31	Upregulation
53353	LRP1B	16557	0.19	0.66	Upregulation
8767	RIPK2	2588	15.69	54.34	Upregulation
734	OSGIN2	4224.11	7.09	24.55	Upregulation
79993	ELOVL7	3851	1.18	4.08	Upregulation
9595	CYTIP	2282	5.94	20.52	Upregulation
1294	COL7A1	9169	22.07	76.21	Upregulation

253980	KCTD13	1859.96	6.19	21.25	Upregulation
3552	IL1A	2947	24.95	85.52	Upregulation
4929	NR4A2	3546	21.87	74.72	Upregulation
100127888	SLCO4A1-AS1	1440	0.88	2.94	Upregulation
2182	ACSL4	5075.69	51.25	170.67	Upregulation
9071	CLDN10	2490	2.14	7.12	Upregulation
728640	FAM133CP	2520	0.77	2.54	Upregulation
768213	MIR671	118	13.13	43.28	Upregulation
26580	BSCL2	1746.89	3.08	10.08	Upregulation
3553	IL1B	1498	149.85	490.11	Upregulation
10211	FLOT1	2025.24	971.22	3176.13	Upregulation
65010	SLC26A6	2522.47	17.13	55.94	Upregulation
4854	NOTCH3	8089	0.69	2.25	Upregulation
390940	PINLYP	938	13.77	44.81	Upregulation
7020	TFAP2A	3508.21	10.04	32.5	Upregulation
101927599	ZNF529-AS1	1193.64	1.57	5.06	Upregulation
56666	PANX2	3066.87	4.09	13.16	Upregulation
3576	CXCL8	1718	1737.3	5586.33	Upregulation
339344	MYPOP	1909	4.14	13.3	Upregulation
283991	UBALD2	1604	17.79	56.98	Upregulation
25764	HYPK	1347.19	2.68	8.58	Upregulation
253982	ASPHD1	1562	5.22	16.59	Upregulation
5122	PCSK1	4824	0.26	0.82	Upregulation
23529	CLCF1	1849.94	6.4	20.17	Upregulation
6372	CXCL6	1677	5.36	16.88	Upregulation
9592	IER2	2088	13.46	42.25	Upregulation
84879	MFSD2A	2157.34	7.48	23.38	Upregulation
342897	NCCRP1	1973	1.17	3.64	Upregulation
282974	STK32C	872.44	2.77	8.53	Upregulation
10252	SPRY1	2486.77	4.29	13.2	Upregulation
54498	SMOX	2240.36	11.35	34.74	Upregulation
644019	CBWD6	1719	0.83	2.53	Upregulation
255043	TMEM86B	1751	1.33	4.04	Upregulation
100874014	NAV2-AS2	853	1.9	5.69	Upregulation
3726	JUNB	1832	46.79	139.77	Upregulation
100133331	LOC100133331	4273	2.98	8.89	Upregulation
8681	JMJD7-PLA2G4B	3379	2.09	6.23	Upregulation
10360	NPM3	904	4.17	12.39	Upregulation
2069	EREG	4628	0.7	2.07	Upregulation
100289561	LOC100289561	1056	1.73	5.1	Upregulation
445329	SULT1A4	1397	4.28	12.53	Upregulation
9586	CREB5	8061.89	1.3	3.8	Upregulation
100133091	LOC100133091	2712	4.86	14.19	Upregulation
5321	PLA2G4A	2886.5	4.77	13.88	Upregulation
283209	PGM2L1	8513	4.39	12.74	Upregulation
386593	CHKB-CPT1B	4293	2.07	6	Upregulation
4773	NFATC2	6850	0.47	1.36	Upregulation
10309	CCNO	1475.15	3.13	9.05	Upregulation

402176	RPL21P44	1797	1.35	3.9	Upregulation
145741	C2CD4A	3461	1.01	2.91	Upregulation
84243	ZDHHC18	3165	3.57	10.24	Upregulation
55151	TMEM38B	3558	1.76	5.02	Upregulation
100874085	NUCB1-AS1	433	6.78	19.28	Upregulation
604	BCL6	3505.81	21.42	60.9	Upregulation
100529145	TEN1-CDK3	3330	0.94	2.67	Upregulation
100137047	JMJD7	1439	3.42	9.67	Upregulation
90865	IL33	2488.66	6.49	18.33	Upregulation
51042	ZNF593	653	6.71	18.94	Upregulation
3487	IGFBP4	2246	21.54	60.41	Upregulation
100506124	LOC100506124	1612	1.31	3.67	Upregulation
100289274	DNAJC3-AS1	2908	0.94	2.63	Upregulation
10046	MAMLD1	4531.76	5.55	15.51	Upregulation
23657	SLC7A11	9648	10.18	28.36	Upregulation
272	AMPD3	4230.65	8.95	24.78	Upregulation
27090	ST6GALNAC4	1692.29	8.37	23.15	Upregulation
10586	MAB21L2	2785	18.67	51.57	Upregulation
5101	PCDH9	4698.64	0.66	1.82	Upregulation
93185	IGSF8	2293.19	12.93	35.39	Upregulation
58472	SQRDL	1874.69	52.09	142.15	Upregulation
7076	TIMP1	931	182.96	497.59	Upregulation
113878	DTX2	2635.52	12.58	34.17	Upregulation
64116	SLC39A8	2870.52	33.14	90.01	Upregulation
11096	ADAMTS5	9680	2.5	6.79	Upregulation
7291	TWIST1	1669	9.19	24.87	Upregulation
94032	CAMK2N2	1360	2.11	5.7	Upregulation
4502	MT2A	466	1326.58	3583.55	Upregulation
55556	ENOSF1	3797.04	9.49	25.6	Upregulation
64374	SIL1	1910.13	14.92	40.14	Upregulation
56971	CEACAM19	2264.04	13.97	37.48	Upregulation
8676	STX11	5509	0.66	1.77	Upregulation
5218	CDK14	4926.09	3.45	9.25	Upregulation
10525	HYOU1	4546.93	51.41	137.39	Upregulation
2171	FABP5	751	35.04	93.56	Upregulation
1803	DPP4	3913	38.43	102.61	Upregulation
4857	NOVA1	2391.22	15.28	40.41	Upregulation
100128252	ZNF667-AS1	1513.44	3.2	8.46	Upregulation
3005	H1F0	2336	5.67	14.96	Upregulation
100507588	TGFBR3L	1256	2.12	5.59	Upregulation
80328	ULBP2	1424	3.9	10.24	Upregulation
1514	CTSL	1571.72	51.98	135.81	Upregulation
113189	CHST14	2213	7.7	20.08	Upregulation
6781	STC1	3897	168.37	438.61	Upregulation
7538	ZFP36	1752	8.2	21.32	Upregulation
1491	CTH	2070.27	1.28	3.32	Upregulation
84444	DOT1L	7455	5.42	14.05	Upregulation
23213	SULF1	5481.32	8.08	20.78	Upregulation

132228	LSMEM2	1583	2.21	5.68	Upregulation
123	PLIN2	2029	41.17	105.62	Upregulation
80728	ARHGAP39	4687.25	1.56	4	Upregulation
10954	PDIA5	1866.27	9.69	24.68	Upregulation
92737	DNER	3272	146.95	374.06	Upregulation
5396	PRRX1	4031.1	9.12	23.17	Upregulation
4211	MEIS1	3198	7.46	18.88	Upregulation
55630	SLC39A4	705.15	5.01	12.67	Upregulation
51303	FKBP11	807.92	10.1	25.45	Upregulation
100506714	NUP50-AS1	1333.42	4.02	10.12	Upregulation
4494	MT1F	465.15	10.08	25.33	Upregulation
27106	ARRDC2	2529.94	7.25	18.19	Upregulation
55117	SLC6A15	4676.09	6.53	16.37	Upregulation
27040	LAT	1677	1.51	3.78	Upregulation
10459	MAD2L2	1165.49	16.9	42.19	Upregulation
11174	ADAMTS6	7289	3.87	9.64	Upregulation
8835	SOCS2	2619.51	5.6	13.92	Upregulation
8110	DPF3	3983.06	3.34	8.3	Upregulation
100506060	SMG1P7	1891	1.76	4.37	Upregulation
9766	SUSD6	5393	23.7	58.77	Upregulation
29107	NXT1	1176	4.81	11.88	Upregulation
114899	C1QTNF3	3624	0.62	1.53	Upregulation
338758	LINC00936	2998	2.31	5.69	Upregulation
4783	NFIL3	2101.64	14.02	34.52	Upregulation
7016	TESK1	2520.45	7.59	18.68	Upregulation
64061	TSPYL2	2833	17.83	43.71	Upregulation
399909	PCNX3	6584	5.09	12.47	Upregulation
100302254	MIR1282	101	64.16	156.98	Upregulation
644172	LOC644172	1762	2.92	7.14	Upregulation
24146	CLDN15	1816.86	2.45	5.98	Upregulation
3656	IRAK2	3469	18.92	46.14	Upregulation
642846	LOC642846	3514	0.87	2.12	Upregulation
112869	SGF29	1160	5.83	14.2	Upregulation
57224	NHSL1	7066.8	0.76	1.85	Upregulation
10019	SH2B3	4661.85	30.14	73.23	Upregulation
55036	CCDC40	3588.51	1.61	3.9	Upregulation
9601	PDIA4	2970	58.51	141.69	Upregulation
2784	GNB3	1760	6.84	16.55	Upregulation
9546	APBA3	2158	4.88	11.79	Upregulation
51635	DHRS7	1404	14.98	36.19	Upregulation
8848	TSC22D1	3330.69	115.46	278.29	Upregulation
101927559	CEBPB-AS1	2973	21.04	50.68	Upregulation
64849	SLC13A3	4069.04	3.35	8.05	Upregulation
57476	GRAMD1B	7636	2.73	6.56	Upregulation
25816	TNFAIP8	2085.08	21.97	52.68	Upregulation
85450	ITPRIP	6631.12	22.84	54.71	Upregulation
159686	CFAP58	3313	0.82	1.96	Upregulation
220213	OTUD1	3125	1.47	3.51	Upregulation

53916	RAB4B	1200	7.85	18.73	Upregulation
102800317	LOC400927-CSNK1E	2997	2.22	5.29	Upregulation
525	ATP6V1B1	1956	1.29	3.07	Upregulation
6158	RPL28	1066.61	795.48	1887.26	Upregulation
8786	RGS11	2407.54	1.16	2.75	Upregulation
91056	AP5B1	6595	5.13	12.16	Upregulation
79897	RPP21	565.59	8.99	21.29	Upregulation
3720	JARID2	5780.78	7.5	17.75	Upregulation
84447	SYVN1	3072.91	23.71	55.97	Upregulation
1051	CEBPB	2113	98.26	231.9	Upregulation
3134	HLA-F	1288.63	43.18	101.77	Upregulation
56605	ERO1B	5070	2.61	6.12	Upregulation
7015	TERT	4018	3.79	8.87	Upregulation
146705	ENTHD2	2308	4.55	10.64	Upregulation
11153	FICD	1651	3.59	8.38	Upregulation
27042	DIEXF	8505	4.7	10.96	Upregulation
51073	MRPL4	1545.93	13.13	30.53	Upregulation
7673	ZNF222	1669.15	2.33	5.41	Upregulation
3439	IFNA1	863	4.63	10.75	Upregulation
22932	POMZP3	1430.97	4.18	9.68	Upregulation
26233	FBXL6	1791.25	2.95	6.83	Upregulation
100506451	RASSF8-AS1	966.64	7.92	18.33	Upregulation
90007	MIDN	3812	13.67	31.63	Upregulation
25973	PARS2	2403	1.53	3.54	Upregulation
54923	LIME1	1399.43	3.77	8.7	Upregulation
27071	DAPP1	2982.04	1.25	2.88	Upregulation
399664	MEX3D	2728.79	2.55	5.87	Upregulation
9718	ECE2	1027	2.72	6.26	Upregulation
8804	CREG1	2048	3.65	8.37	Upregulation
55009	C19orf24	912	8.86	20.3	Upregulation
53838	C11orf24	2120	10.68	24.45	Upregulation
64788	LMF1	2620	1.8	4.12	Upregulation
4294	MAP3K10	3453	4.42	10.1	Upregulation
7050	TGIF1	1587.36	63.8	145.76	Upregulation
80761	UPK3B	1620.57	2.12	4.84	Upregulation
51025	PAM16	600	5.35	12.19	Upregulation
728130	NUTM2D	5543	0.7	1.59	Upregulation
100170841	C17orf96	3232	0.97	2.2	Upregulation
30850	CDR2L	3546	6.4	14.48	Upregulation
64773	PCED1A	1964.16	11.37	25.67	Upregulation
124976	SPNS2	4349	1.75	3.95	Upregulation
23136	EPB41L3	3959.42	32.43	73.16	Upregulation
10043	TOM1	2384.13	12.45	28.08	Upregulation
23223	RRP12	4449	7.69	17.33	Upregulation
10076	PTPRU	5598.83	4.24	9.55	Upregulation
124402	UBALD1	1430	12.13	27.18	Upregulation
64208	POPDC3	1479.5	3.45	7.73	Upregulation

144097	C11orf84	2055	11.22	25.13	Upregulation
83858	ATAD3B	2605	4.77	10.68	Upregulation
116138	KLHDC3	2029	17.19	38.48	Upregulation
7477	WNT7B	3928	4.35	9.73	Upregulation
26470	SEZ6L2	3425.66	42.79	95.39	Upregulation
388759	C1orf229	2258	1.59	3.54	Upregulation
57214	CEMIP	7130.95	86.05	191.52	Upregulation
3624	INHBA	2175	94.43	209.73	Upregulation
84926	SPRYD3	2949	6.75	14.97	Upregulation
54972	TMEM132A	3518.52	80.04	177.17	Upregulation
84002	B3GNT5	4131	1.92	4.24	Upregulation
27338	UBE2S	1207	63.37	139.82	Upregulation
9945	GFPT2	3115	9.78	21.55	Upregulation
1047	CLGN	2744	33.24	73.15	Upregulation
10740	RFPL1S	5125	0.6	1.32	Upregulation
118813	ZFYVE27	2733.58	16.17	35.51	Upregulation
64118	DUS1L	1904	15.73	34.52	Upregulation
101929532	LOC101929532	3609	14.74	32.3	Upregulation
2669	GEM	2139.43	7.09	15.53	Upregulation
4323	MMP14	3610	494.69	1083.22	Upregulation
27079	RPUSD2	2048.72	2.28	4.98	Upregulation
6520	SLC3A2	1926.86	41.27	90.02	Upregulation
10549	PRDX4	921	46.08	100.46	Upregulation
112970	KTI12	1712	2.71	5.89	Upregulation
23645	PPP1R15A	2399	29.16	63.32	Upregulation
25893	TRIM58	5158	0.76	1.65	Upregulation
2888	GRB14	2277.38	13.64	29.6	Upregulation
5971	RELB	2297	13.09	28.38	Upregulation
2303	FOXC2	1683	12.21	26.47	Upregulation
10572	SIVA1	790	4.94	10.7	Upregulation
65249	ZSWIM4	4339	5.48	11.86	Upregulation
6478	SIAH2	2632	5.98	12.94	Upregulation
4814	NINJ1	1297	69.68	150.65	Upregulation
117581	TWIST2	1401.07	3.09	6.66	Upregulation
90317	ZNF616	3070	1.1	2.37	Upregulation
84068	SLC10A7	3811.51	2.3	4.95	Upregulation
81848	SPRY4	4995.04	4.42	9.5	Upregulation
605	BCL7A	3761.86	1.15	2.47	Upregulation
56257	MEPCE	2676.46	16.6	35.65	Upregulation
166	AES	1687.3	93.56	200.65	Upregulation
57026	PDXP	2072	6.96	14.87	Upregulation
79856	SNX22	3615.17	59.24	126.5	Upregulation
1604	CD55	2101.99	62.06	132.47	Upregulation
415116	PIM3	2410	13.51	28.82	Upregulation
100532735	INO80B-WBP1	2112	11.94	25.45	Upregulation
90141	EFCAB11	2804.88	3.36	7.16	Upregulation
654	BMP6	3105	15.74	33.54	Upregulation
5187	PER1	4717	4.53	9.65	Upregulation

4791	NFKB2	3143.86	68.34	145.43	Upregulation
89927	C16orf45	2232.78	10.26	21.83	Upregulation
57647	DHX37	4568	2.85	6.06	Upregulation
7461	CLIP2	5461.44	19.14	40.68	Upregulation
11054	OGFR	2423	28	59.44	Upregulation
2296	FOXC1	3452	2.6	5.51	Upregulation
694	BTG1	4704	61.11	129.27	Upregulation
654364	NME1-NME2	1095.97	6.66	14.08	Upregulation
126298	IRGQ	9703	8.84	18.67	Upregulation
10514	MYBBP1A	4344.62	8.81	18.56	Upregulation
50512	PODXL2	2209	8.76	18.45	Upregulation
144717	FAM109A	3168	1.24	2.61	Upregulation
1843	DUSP1	2040	58.71	123.51	Upregulation
79174	CRELD2	1456.2	15.44	32.46	Upregulation
10195	ALG3	1556.58	14.01	29.43	Upregulation
133308	SLC9B2	2820.21	1.82	3.82	Upregulation
55170	PRMT6	2665	3.54	7.43	Upregulation
9846	GAB2	6068.24	6.9	14.47	Upregulation
284040	CDRT4	2515	7.93	16.63	Upregulation
11173	ADAMTS7	5552	6.29	13.19	Upregulation
9120	SLC16A6	3844.06	48.16	100.97	Upregulation
1646	AKR1C2	2951.42	0.83	1.74	Upregulation
1649	DDIT3	991.56	22.38	46.84	Upregulation
644096	SDHAF1	1147	5.78	12.09	Upregulation
84844	PHF5A	1105	12.39	25.91	Upregulation
121551	BTBD11	4175.96	1.79	3.74	Upregulation
56905	C15orf39	4443	6.67	13.93	Upregulation
54620	FBXL19	3796.77	6.35	13.24	Upregulation
2643	GCH1	2925.6	6.59	13.73	Upregulation
85302	FBF1	4696	0.97	2.02	Upregulation
6415	SEPW1	893	12.92	26.9	Upregulation
9275	BCL7B	1754.39	13.64	28.39	Upregulation
8836	GGH	1505	9.23	19.21	Upregulation
140885	SIRPA	3883.22	26.11	54.31	Upregulation
5055	SERPINB2	1924.17	223.13	464.08	Upregulation
7097	TLR2	3601.02	1.54	3.2	Upregulation
842	CASP9	2803.12	1.96	4.07	Upregulation
6583	SLC22A4	2214	7.47	15.51	Upregulation
4595	MUTYH	1892.44	2.74	5.68	Upregulation
221491	C6orf1	1005.72	10.91	22.6	Upregulation
2043	EPHA4	6407.84	6.83	14.14	Upregulation
221545	C6orf136	1497.5	5.57	11.53	Upregulation
29952	DPP7	1633	53.98	111.7	Upregulation
80148	PQLC1	2512.05	4.5	9.31	Upregulation
84446	BRSK1	3109	14.81	30.63	Upregulation
10482	NXF1	3007.1	25.1	51.89	Upregulation
10082	GPC6	7114	7.72	15.94	Upregulation
3570	IL6R	4264.33	3.43	7.08	Upregulation

80324	PUS1	1700.51	3.94	8.13	Upregulation
83667	SESN2	3547	4.45	9.18	Upregulation
3399	ID3	1252	29.96	61.79	Upregulation
1802	DPH2	2328.85	4.7	9.69	Upregulation
23184	MESDC2	3539.28	11.37	23.41	Upregulation
54910	SEMA4C	3585	7.87	16.19	Upregulation
1356	CP	4273.7	4.13	8.49	Upregulation
653268	AGAP7P	2423	3.87	7.93	Upregulation
387856	CCDC184	2380	1.85	3.79	Upregulation
23277	CLUH	5252	7.76	15.89	Upregulation
5886	RAD23A	1797.09	52.85	108.15	Upregulation
11228	RASSF8	5545.45	61.33	125.47	Upregulation
5034	P4HB	2596	293.8	600.45	Upregulation
8877	SPHK1	1922.61	5.95	12.16	Upregulation
84163	GTF2IRD2	2280.4	5.3	10.81	Upregulation
84221	SPATC1L	1244.33	7.64	15.57	Upregulation
3032	HADHB	2196	37.19	75.69	Upregulation
8875	VNN2	1977.61	2.85	5.8	Upregulation
84957	RELT	3463.94	2.21	4.49	Upregulation
29990	PILRB	1438	15.48	31.4	Upregulation
55022	PID1	2637.81	41.57	84.28	Upregulation
79581	SLC52A2	1958.75	12.74	25.8	Upregulation
22927	HABP4	2681	4.38	8.87	Upregulation
4157	MC1R	3115	1.71	3.46	Upregulation
5589	PRKCSH	2114.9	118.29	239.2	Upregulation
9704	DHX34	4390	2.73	5.51	Upregulation
8527	DGKD	6236.92	6.48	13.05	Upregulation
10817	FRS3	2198	2.35	4.73	Upregulation
8131	NPRL3	2883.93	7.14	14.36	Upregulation
51477	ISYNA1	2129	1.99	4	Upregulation
7873	MANF	993	77.66	156.08	Upregulation
9136	RRP9	1590	5.13	10.31	Upregulation
10957	PNRC1	2133	33.11	66.53	Upregulation
283871	PGP	3136	3.51	7.05	Upregulation
84080	ENKD1	1628	6.5	13.05	Upregulation
7866	IFRD2	2129	6.03	12.1	Upregulation
114987	WDR31	4883.76	6.43	12.87	Upregulation
3105	HLA-A	1616.43	1188.18	2377.03	Upregulation
92002	FAM58A	1291.11	3.58	7.16	Upregulation
196483	EEF2KMT	2279.08	6.07	12.14	Upregulation
284440	LINC00663	2385	2.12	4.24	Upregulation
8928	FOXH1	2193	3.26	6.52	Upregulation
100534592	URGCP-MRPS24	838	4.81	0.01	Downregulation
728806	NSFP1	587	2.72	0.01	Downregulation
5342	PLGLB2	3016	2.48	0.01	Downregulation
727866	FAM156B	1701	2.05	0.01	Downregulation
10170	DHRS9	1741.3	2	0.01	Downregulation
100505865	LINC00920	913	1.42	0.01	Downregulation

100526772	TMEM110-MUSTN1	1459	1.2	0.01	Downregulation
202658	TRIM39-RPP21	1567	1.2	0.01	Downregulation
103171574	LOC103171574	1311	1.16	0.01	Downregulation
388436	LOC388436	1735	1.09	0.01	Downregulation
440243	GOLGA6L22	3806	0.9	0.01	Downregulation
554282	FAM72C	2338	0.83	0.01	Downregulation
157570	ESCO2	3376	0.81	0.01	Downregulation
101928994	LOC101928994	1933	0.78	0.01	Downregulation
100529215	ZNF559-ZNF177	2723.07	0.78	0.01	Downregulation
100533483	DYX1C1-CCPG1	4838	0.71	0.01	Downregulation
150094	SIK1	4706	0.69	0.01	Downregulation
1134	CHRNA1	2019	0.68	0.01	Downregulation
286749	STON1-GTF2A1L	3807.14	0.62	0.01	Downregulation
338382	RAB7B	2955	0.57	0.01	Downregulation
2359	FPR3	2643	0.48	0.01	Downregulation
57105	CYSLTR2	2954	0.43	0.01	Downregulation
728047	GOLGA8O	5189	2.04	0.07	Downregulation
399844	LINC01002	4874	0.26	0.01	Downregulation
22914	KLRK1	1606	4.68	0.22	Downregulation
136853	SSC4D	2806	3.03	0.15	Downregulation
632	BGLAP	760	5.41	0.27	Downregulation
9075	CLDN2	2932	0.79	0.04	Downregulation
80099	C7orf69	679	3.14	0.16	Downregulation
6518	SLC2A5	2454	0.77	0.04	Downregulation
2307	FOXS1	1353	1.49	0.08	Downregulation
81849	ST6GALNAC5	5110	0.37	0.02	Downregulation
100526739	APITD1-CORT	1383	1.83	0.11	Downregulation
4605	MYBL2	2713	0.62	0.04	Downregulation
195828	ZNF367	3714	0.45	0.03	Downregulation
11262	SP140	2928	1.2	0.08	Downregulation
5136	PDE1A	3993.5	0.75	0.05	Downregulation
56256	SERTAD4	2028	3.48	0.26	Downregulation
374462	PTPRQ	8066	0.39	0.03	Downregulation
9582	APOBEC3B	1485	2.02	0.16	Downregulation
5923	RASGRF1	4860	0.47	0.04	Downregulation
5493	PPL	6256	4.97	0.43	Downregulation
56062	KLHL4	5880	2.88	0.25	Downregulation
57493	HEG1	9156	97.29	8.54	Downregulation
5570	PKIB	2012	14.34	1.26	Downregulation
114800	CCDC85A	3983	0.89	0.08	Downregulation
29943	PADI1	3847	0.54	0.05	Downregulation
653820	FAM72B	1861	1.39	0.13	Downregulation
5028	P2RY1	6802	0.95	0.09	Downregulation
10100	TSPAN2	3143	0.73	0.07	Downregulation
6563	SLC14A1	3930.3	30.02	2.93	Downregulation
29995	LMCD1	1877.25	2.22	0.22	Downregulation
219790	RTKN2	5840.47	2.34	0.24	Downregulation
58538	MPP4	2475	4.5	0.47	Downregulation

55872	PBK	2102	2.67	0.3	Downregulation
55247	NEIL3	2402	0.79	0.09	Downregulation
51704	GPRC5B	2941	9.62	1.1	Downregulation
168507	PKD1L1	9075	0.25	0.03	Downregulation
8436	SDPR	3265	0.83	0.1	Downregulation
6326	SCN2A	8571	1.16	0.14	Downregulation
100506498	LOC100506498	1202	2.22	0.27	Downregulation
57082	CASC5	7607	0.57	0.07	Downregulation
3952	LEP	3444	0.65	0.08	Downregulation
100506621	LINC01279	5176	28.62	3.61	Downregulation
8302	KLRC4	928	2.72	0.35	Downregulation
10800	CYSLTR1	2693	1.24	0.16	Downregulation
64220	STRA6	2802	0.82	0.11	Downregulation
11130	ZWINT	1546	2.01	0.27	Downregulation
1264	CNN1	1646	1.41	0.19	Downregulation
283554	GPR137C	3908	0.59	0.08	Downregulation
1288	COL4A6	6552	0.44	0.06	Downregulation
643246	MAP1LC3B2	832	2.38	0.33	Downregulation
221150	SKA3	2808	1.36	0.19	Downregulation
51514	DTL	4381	1.5	0.21	Downregulation
374897	SBSN	1998	1.8	0.26	Downregulation
170688	NUDT4P2	3838	2.12	0.31	Downregulation
9052	GPRC5A	2856	37.22	5.55	Downregulation
84189	SLITRK6	4199	0.67	0.1	Downregulation
4288	MKI67	11715.37	4.81	0.72	Downregulation
283768	GOLGA8G	5282	1.12	0.17	Downregulation
7272	TTK	3010	2.3	0.35	Downregulation
400221	FLJ22447	1386	5.06	0.77	Downregulation
10733	PLK4	3693	1.31	0.2	Downregulation
9787	DLGAP5	3035.14	2.21	0.34	Downregulation
730101	LOC730101	3616.7	38.63	5.97	Downregulation
23555	TSPAN15	1726	2.76	0.43	Downregulation
11346	SYNPO	5329.26	8.05	1.26	Downregulation
287	ANK2	7988	1.93	0.31	Downregulation
10721	POLQ	8787	0.37	0.06	Downregulation
6319	SCD	5473	72.75	11.86	Downregulation
81553	FAM49A	4702	1.22	0.2	Downregulation
53844	COPG2IT1	3463	17.18	2.83	Downregulation
6941	TCF19	3060	1.03	0.17	Downregulation
100422558	SMG1P6	3132	1.21	0.2	Downregulation
503637	DUXAP8	2107	1.45	0.24	Downregulation
57484	RNF150	4661	1.5	0.25	Downregulation
27063	ANKRD1	1994	294.26	49.4	Downregulation
781	CACNA2D1	7583	39.49	6.67	Downregulation
1296	COL8A2	4397	1.1	0.19	Downregulation
2357	FPR1	1334	6.81	1.19	Downregulation
115701	ALPK2	7303	3.12	0.55	Downregulation

4900	NRGN	1235	3.88	0.69	Downregulation
51313	FAM198B	4809	7.08	1.26	Downregulation
389336	C5orf46	587	7.33	1.31	Downregulation
51512	GTSE1	3128	2.07	0.37	Downregulation
4232	MEST	2424.78	95.78	17.13	Downregulation
5137	PDE1C	4431.9	10.45	1.9	Downregulation
10396	ATP8A1	8225	0.82	0.15	Downregulation
288	ANK3	9356	2.24	0.41	Downregulation
1301	COL11A1	7291	8.4	1.54	Downregulation
5176	SERPINF1	1552	1.84	0.34	Downregulation
283104	SBF2-AS1	2708	25.8	4.83	Downregulation
8515	ITGA10	5054.51	8.49	1.59	Downregulation
10148	EBI3	1149	4.46	0.84	Downregulation
6241	RRM2	3284	9.75	1.87	Downregulation
3791	KDR	6055	2.24	0.43	Downregulation
84978	FRMD5	5077	2.03	0.39	Downregulation
6274	S100A3	738	4.57	0.88	Downregulation
9099	USP2	2933	2.02	0.39	Downregulation
3823	KLRC3	930.85	7.29	1.42	Downregulation
65061	CDK15	3258.84	0.92	0.18	Downregulation
10112	KIF20A	3471	2.29	0.45	Downregulation
25802	LMOD1	3967	12.32	2.44	Downregulation
6328	SCN3A	8994	1.21	0.24	Downregulation
103021295	LOC103021295	464	7.71	1.53	Downregulation
55723	ASF1B	1746	1.51	0.3	Downregulation
10769	PLK2	2820.93	321.63	63.9	Downregulation
727936	GXYLT2	1547	3.77	0.75	Downregulation
3822	KLRC2	1223	14.7	2.94	Downregulation
306	ANXA3	1634	22.92	4.59	Downregulation
9369	NRXN3	8826.93	2.09	0.42	Downregulation
57650	KIAA1524	4284	1.19	0.24	Downregulation
145508	CEP128	4461	0.94	0.19	Downregulation
4645	MYO5B	9520	0.69	0.14	Downregulation
1063	CENPF	10316	3.77	0.77	Downregulation
143686	SESN3	9443	0.93	0.19	Downregulation
388610	TRNP1	1949	3.41	0.7	Downregulation
54538	ROBO4	3653	1.12	0.23	Downregulation
1285	COL4A3	8114	1.4	0.29	Downregulation
220965	FAM13C	3336.73	2.1	0.44	Downregulation
57088	PLSCR4	3208.97	14.56	3.09	Downregulation
151354	FAM84A	6366	0.42	0.09	Downregulation
2491	CENPI	3263	0.83	0.18	Downregulation
338596	ST8SIA6	1398	2.81	0.61	Downregulation
51196	PLCE1	6629.68	4.16	0.91	Downregulation
26024	PTCD1	5505	2.95	0.65	Downregulation
387758	FIBIN	3024	3.29	0.73	Downregulation
100874212	MYCBP2-AS1	517	5.81	1.29	Downregulation
3730	ANOS1	6314	6.53	1.45	Downregulation

100507421	TMEM178B	10558	0.63	0.14	Downregulation
1286	COL4A4	10341	2.41	0.54	Downregulation
222696	ZSCAN23	3178	0.89	0.2	Downregulation
7098	TLR3	3057	2.88	0.65	Downregulation
5507	PPP1R3C	2576	10.63	2.4	Downregulation
55304	SPTLC3	3855	4.77	1.08	Downregulation
22998	LIMCH1	5985.75	106.06	24.21	Downregulation
3352	HTR1D	2857	14.13	3.24	Downregulation
8470	SORBS2	4490.71	7.31	1.69	Downregulation
23551	RASD2	3047	2.2	0.51	Downregulation
84561	SLC12A8	3449	9.39	2.18	Downregulation
5318	PKP2	4420.51	8.91	2.08	Downregulation
652	BMP4	1873.83	53.6	12.6	Downregulation
113130	CDCA5	2599	1.53	0.36	Downregulation
654342	LOC654342	613	6.09	1.44	Downregulation
259232	NALCN	6923	5.02	1.19	Downregulation
55165	CEP55	2534	5.55	1.32	Downregulation
11004	KIF2C	2799	3.91	0.93	Downregulation
259266	ASPM	10578.74	2.06	0.49	Downregulation
63940	GPSM3	1501	2.05	0.49	Downregulation
2195	FAT1	14773	29.18	7.02	Downregulation
79026	AHNAK	16526.76	61.51	14.91	Downregulation
64399	HHIP	3555	14.88	3.61	Downregulation
1462	VCAN	7737.81	407.28	98.98	Downregulation
983	CDK1	1912.84	5.43	1.32	Downregulation
1382	CRABP2	1088	13.62	3.34	Downregulation
1906	EDN1	2109	5.69	1.4	Downregulation
7480	WNT10B	2370	1.42	0.35	Downregulation
80144	FRAS1	15643	3.21	0.8	Downregulation
100144602	EPHA5-AS1	1198	4.28	1.07	Downregulation
57158	JPH2	4301.61	1.36	0.34	Downregulation
8416	ANXA9	1843	1.83	0.46	Downregulation
41	ASIC1	3847.98	20.02	5.04	Downregulation
54704	PDP1	4247	27.67	6.97	Downregulation
5414	SEPT4	1679	2.23	0.57	Downregulation
25894	PLEKHG4	4590.28	4.24	1.09	Downregulation
83540	NUF2	1843	3.34	0.86	Downregulation
1824	DSC2	5260	4.14	1.07	Downregulation
1836	SLC26A2	8082	17.05	4.41	Downregulation
400966	RGPD1	6711	0.73	0.19	Downregulation
57181	SLC39A10	5337	17.05	4.44	Downregulation
643314	KIAA0754	6979	7.13	1.86	Downregulation
1062	CENPE	8267	0.92	0.24	Downregulation
5922	RASA2	5647.2	35.71	9.37	Downregulation
100652770	DSG2-AS1	2000	2.21	0.58	Downregulation
80312	TET1	9601	0.8	0.21	Downregulation

23500	DAAM2	6191	2.74	0.72	Downregulation
5352	PLOD2	4063.51	332.62	87.46	Downregulation
8869	ST3GAL5	2262	8.6	2.27	Downregulation
55075	UACA	6881.47	44.33	11.71	Downregulation
3866	KRT15	1861	1.93	0.51	Downregulation
116028	RMI2	1228	4.25	1.13	Downregulation
342184	FMN1	12521	1.39	0.37	Downregulation
54762	GRAMD1C	3770	5.15	1.38	Downregulation
4306	NR3C2	5915	11.56	3.11	Downregulation
2044	EPHA5	7560.34	7.11	1.92	Downregulation
51200	CPA4	2817	94.57	25.68	Downregulation
170689	ADAMTS15	5676	12.25	3.33	Downregulation
143872	ARHGAP42	4752	1.03	0.28	Downregulation
9672	SDC3	5121	13.86	3.78	Downregulation
116441	TM4SF18	3731.14	6.85	1.87	Downregulation
5787	PTPRB	10663	4.46	1.22	Downregulation
79801	SHCBP1	3249	2.47	0.68	Downregulation
3161	HMMR	3141.03	1.34	0.37	Downregulation
158471	PRUNE2	5543.1	13.98	3.9	Downregulation
151887	CCDC80	4711.81	131.42	36.73	Downregulation
10535	RNASEH2A	1148	3.63	1.02	Downregulation
353189	SLCO4C1	5334	0.96	0.27	Downregulation
9697	TRAM2	7065	161.44	45.44	Downregulation
397	ARHGDIB	1216	7.46	2.1	Downregulation
101927204	LOC101927204	2247	2.59	0.73	Downregulation
100506835	LINC00840	1974	9.24	2.62	Downregulation
653464	SRGAP2C	3347	3.03	0.86	Downregulation
1903	S1PR3	4404	21.38	6.07	Downregulation
4171	MCM2	3504	3.13	0.89	Downregulation
3756	KCNH1	8025	0.49	0.14	Downregulation
100271836	SMG1P3	5052	4.19	1.2	Downregulation
165215	FAM171B	3557	4.5	1.29	Downregulation
64359	NXN	2742.04	13.98	4.03	Downregulation
4147	MATN2	4178.13	6.98	2.02	Downregulation
10395	DLC1	5908.7	20.33	5.89	Downregulation
29091	STXBP6	3776	1.24	0.36	Downregulation
3433	IFIT2	3505	58.61	17.04	Downregulation
341	APOC1	464	11.57	3.38	Downregulation
301	ANXA1	1556	104.85	30.73	Downregulation
3750	KCND1	4720	3.84	1.13	Downregulation
55137	FIGN	4535	1.63	0.48	Downregulation
81606	LBH	2956	60.31	17.8	Downregulation
8727	CTNNAL1	2573	43.93	12.99	Downregulation
5357	PLS1	3720	4.61	1.37	Downregulation
10635	RAD51AP1	2218	2.22	0.66	Downregulation
8654	PDE5A	6863.02	4.63	1.38	Downregulation
5947	RBP1	963.04	37.3	11.14	Downregulation
84301	DDI2	2680	2.24	0.67	Downregulation

80380	PDCD1LG2	2418	11.6	3.47	Downregulation
100381270	ZBED6	2940	11.9	3.56	Downregulation
11098	PRSS23	3669.78	340.15	102.11	Downregulation
171024	SYNPO2	5755.14	1.56	0.47	Downregulation
100526836	BLOC1S5-TXNDC5	3352	4.38	1.32	Downregulation
25890	ABI3BP	4488	7.3	2.2	Downregulation
388886	LRRC75B	1240	3.7	1.12	Downregulation
9076	CLDN1	3452	39.83	12.07	Downregulation
554236	DPY19L2P1	2827	2.57	0.78	Downregulation
57452	GALNT16	3148	3.16	0.96	Downregulation
1117	CHI3L2	1486	12.2	3.71	Downregulation
84803	GPAT3	2640	2.07	0.63	Downregulation
3685	ITGAV	6805.23	56.16	17.11	Downregulation
7083	TK1	1616	2.55	0.78	Downregulation
6857	SYT1	4517.62	32.53	9.96	Downregulation
56956	LHX9	2515	1.63	0.5	Downregulation
151246	SGO2	4029	1.43	0.44	Downregulation
101241892	NPTN-IT1	2606	2.21	0.68	Downregulation
83593	RASSF5	3531	1.62	0.5	Downregulation
26509	MYOF	6859.48	113.8	35.18	Downregulation
1794	DOCK2	6117	0.87	0.27	Downregulation
728554	LOC728554	1827	3.24	1.01	Downregulation
65065	NBEAL1	9058	1.7	0.53	Downregulation
80034	CSRNP3	11687	0.64	0.2	Downregulation
169611	OLFML2A	6307.21	28.21	8.83	Downregulation
79739	TTLL7	3648	3.29	1.03	Downregulation
11010	GLIPR1	3924	43.08	13.5	Downregulation
7006	TEC	3620	1.18	0.37	Downregulation
785	CACNB4	7793	1.37	0.43	Downregulation
100750246	HIF1A-AS1	652	24.54	7.72	Downregulation
80010	RMI1	3508	1.52	0.48	Downregulation
11113	CIT	8708	1.96	0.62	Downregulation
27254	CSDC2	2545	3.16	1	Downregulation
157285	SGK223	4726	1.32	0.42	Downregulation
25837	RAB26	1641	9.93	3.16	Downregulation
100132406	NBPF10	10916	13.6	4.33	Downregulation
57514	ARHGAP31	8096	3.14	1	Downregulation
386618	KCTD4	2133	6.02	1.92	Downregulation
3690	ITGB3	4894	0.94	0.3	Downregulation
701	BUB1B	3749	1.47	0.47	Downregulation
9348	NDST3	5961	0.78	0.25	Downregulation
51232	CRIM1	5628	189.86	60.98	Downregulation
283234	CCDC88B	4932	0.99	0.32	Downregulation
22874	PLEKHA6	7434	2.53	0.82	Downregulation
3572	IL6ST	8954.54	79.66	25.85	Downregulation
117248	GALNT15	3626.36	5.53	1.8	Downregulation
79627	OGFRL1	1755	35.76	11.66	Downregulation
23397	NCAPH	5806	0.92	0.3	Downregulation

6876	TAGLN	1219.14	73.27	23.94	Downregulation
7039	TGFA	4323.09	2.87	0.94	Downregulation
699	BUB1	3562.96	3.19	1.05	Downregulation
64084	CLSTN2	4882	19.68	6.48	Downregulation
5920	RARRES3	779	32.88	10.85	Downregulation
54843	SYTL2	2510.86	5.33	1.76	Downregulation
220979	C10orf25	928	7.66	2.55	Downregulation
100532736	MINOS1-NBL1	2071.85	26.63	8.87	Downregulation
64859	NABP1	3610.62	18.3	6.1	Downregulation
27124	INPP5J	3295.83	2.28	0.76	Downregulation
1647	GADD45A	1336.85	29.65	9.93	Downregulation
30818	KCNIP3	2735	9.47	3.18	Downregulation
9414	TJP2	4625.8	7.27	2.45	Downregulation
114794	ELFN2	8379	5.03	1.7	Downregulation
1953	MEGF6	7450	20.99	7.12	Downregulation
2861	GPR37	3815	1.12	0.38	Downregulation
54206	ERRFI1	3144	73.77	25.17	Downregulation
29128	UHRF1	4339	3.04	1.04	Downregulation
6095	RORA	10998	0.76	0.26	Downregulation
90102	PHLDB2	5934.89	27.85	9.55	Downregulation
57460	PPM1H	6201	1.28	0.44	Downregulation
1756	DMD	4591	4.42	1.52	Downregulation
631	BFSP1	2200.28	2.35	0.81	Downregulation
100131315	LOC100131315	585	15.84	5.46	Downregulation
51421	AMOTL2	5004.54	49.99	17.26	Downregulation
84078	KBTBD7	3008	2.54	0.88	Downregulation
26031	OSBPL3	6464.75	12.8	4.44	Downregulation
115265	DDIT4L	2649	2.85	0.99	Downregulation
22941	SHANK2	8057.7	1.64	0.57	Downregulation
30061	SLC40A1	3381	25.05	8.71	Downregulation
101929690	LINC01583	2568	3.19	1.11	Downregulation
157638	FAM84B	5503	6.35	2.21	Downregulation
805	CALM2	1316.94	875.32	304.84	Downregulation
2589	GALNT1	3852	151.27	52.69	Downregulation
79940	LINC00472	9499.99	4.59	1.6	Downregulation
2304	FOXE1	3473	8.19	2.86	Downregulation
7357	UGCG	4028	226.57	79.39	Downregulation
8693	GALNT4	5408	3.56	1.25	Downregulation
84832	ANKRD36BP1	1879	3.52	1.24	Downregulation
4008	LMO7	7234.77	35.8	12.62	Downregulation
26298	EHF	5397	3.58	1.27	Downregulation
57523	NYNRIN	7857	1.63	0.58	Downregulation
79899	PRR5L	3856.29	7.08	2.52	Downregulation
23052	ENDOD1	4693	8.22	2.93	Downregulation
8218	CLTCL1	5484.24	5.32	1.9	Downregulation
23233	EXOC6B	2594	7	2.5	Downregulation
2074	ERCC6	7984.79	64.14	22.96	Downregulation
57697	FANCM	7060	0.81	0.29	Downregulation

414236	C10orf55	2516	7.18	2.58	Downregulation
414260	LINC00619	1131	11.81	4.25	Downregulation
4015	LOX	4740.44	169.32	61.13	Downregulation
54947	LPCAT2	5395	104.7	37.83	Downregulation
63979	FIGNL1	3417.89	2.02	0.73	Downregulation
134	ADORA1	2782	1.77	0.64	Downregulation
440823	MIAT	10012.59	15.34	5.56	Downregulation
220594	USP32P2	4859	2.81	1.02	Downregulation
5358	PLS3	3390.84	101.2	36.8	Downregulation
8507	ENC1	4484.15	8.74	3.18	Downregulation
100874207	ST3GAL6-AS1	769	6.21	2.26	Downregulation
55166	CENPQ	1753	3.62	1.32	Downregulation
222865	TMEM130	2871.89	6.14	2.24	Downregulation
5740	PTGIS	5603	6.74	2.46	Downregulation
147	ADRA1B	2272	3.42	1.25	Downregulation
9493	KIF23	3609.91	4.35	1.59	Downregulation
84620	ST6GAL2	5091.18	3.58	1.31	Downregulation
646	BNC1	4627	1.53	0.56	Downregulation
100294145	LOC100294145	3668.27	3.66	1.34	Downregulation
93663	ARHGAP18	3503	43.93	16.12	Downregulation
6414	SEPP1	2252	2.15	0.79	Downregulation
1520	CTSS	4107	16.57	6.09	Downregulation
84698	CAPS2	3075.89	3.89	1.43	Downregulation
22836	RHOBTB3	5566	52.72	19.4	Downregulation
130814	PQLC3	1842.45	5.12	1.89	Downregulation
1734	DIO2	6177.61	8.06	2.98	Downregulation
9928	KIF14	6881	1.27	0.47	Downregulation
221091	LRRN4CL	2600	4.35	1.61	Downregulation
152007	GLIPR2	1952	5.22	1.94	Downregulation
254827	NAALADL2	4912	1.21	0.45	Downregulation
55635	DEPDC1	4607	1.45	0.54	Downregulation
134429	STARD4	4213.02	28.1	10.47	Downregulation
83716	CRISPLD2	4607	1.15	0.43	Downregulation
4012	LNPEP	4404.43	5.8	2.18	Downregulation
4603	MYBL1	5084.87	37.47	14.12	Downregulation
100288911	LOC100288911	733	10.99	4.15	Downregulation
114902	C1QTNF5	1361	4.74	1.79	Downregulation
340719	NANOS1	4036	7.44	2.81	Downregulation
493	ATP2B4	8766.61	50.28	19.01	Downregulation
8411	EEA1	8019	5.13	1.94	Downregulation
79071	ELOVL6	3210.45	12.7	4.82	Downregulation
3717	JAK2	5285	7.78	2.96	Downregulation
8450	CUL4B	5198	40.55	15.45	Downregulation
22974	TPX2	3685	6.98	2.66	Downregulation
201562	HACD2	1187	9.89	3.77	Downregulation
7402	UTRN	12436	7.52	2.87	Downregulation
1287	COL4A5	6523	48.16	18.41	Downregulation

5087	PBX1	5817.64	22.22	8.51	Downregulation
1033	CDKN3	906	7.73	2.97	Downregulation
3833	KIFC1	2721	2.68	1.03	Downregulation
1122	CHML	7082	3.3	1.27	Downregulation
83937	RASSF4	2509	18.03	6.94	Downregulation
25780	RASGRP3	4532.85	1.48	0.57	Downregulation
991	CDC20	1697	4.05	1.56	Downregulation
162394	SLFN5	4660	20.82	8.02	Downregulation
56944	OLFML3	1890.35	7.37	2.84	Downregulation
5021	OXTR	4361	7.68	2.96	Downregulation
11217	AKAP2	6815	34.52	13.31	Downregulation
24137	KIF4A	4504	1.27	0.49	Downregulation
27115	PDE7B	5387	13.1	5.06	Downregulation
651746	ANKRD33B	9333	0.62	0.24	Downregulation
122953	JDP2	3793	1.29	0.5	Downregulation
8490	RGS5	5284.06	7.81	3.03	Downregulation
79646	PANK3	3404	8.6	3.34	Downregulation
122786	FRMD6	4504.22	43.15	16.77	Downregulation
10592	SMC2	6030.96	5.53	2.15	Downregulation
220	ALDH1A3	3508.21	73.08	28.42	Downregulation
83872	HMCN1	18212	4.06	1.58	Downregulation
79054	TRPM8	5621	1.64	0.64	Downregulation
105751187	LRP1-AS	655	9.37	3.67	Downregulation
256691	MAMDC2	3618	15.89	6.23	Downregulation
4001	LMNB1	2621.72	5.19	2.04	Downregulation
389136	VGLL3	8153.84	7.27	2.86	Downregulation
648	BMI1	3435	7.64	3.02	Downregulation
51065	RPS27L	1084	195.85	77.42	Downregulation
2744	GLS	4702.31	35.96	14.22	Downregulation
100129792	CCDC152	3491	3.54	1.4	Downregulation
3977	LIFR	10107	2.4	0.95	Downregulation
2185	PTK2B	4217.32	6.31	2.5	Downregulation
9223	MAGI1	7062	4.87	1.93	Downregulation
106635534	SNORA104	139	72.47	28.86	Downregulation
9249	DHRS3	1653.79	27.83	11.12	Downregulation
64115	C10orf54	4774	3.6	1.44	Downregulation
168667	BMPER	5031	7.75	3.1	Downregulation
2202	EFEMP1	3060.76	319.13	127.77	Downregulation
8460	TPST1	2062	23.55	9.44	Downregulation
5129	CDK18	3142.57	12.48	5.02	Downregulation
957	ENTPD5	2064	2.65	1.07	Downregulation
4286	MITF	4362.04	9.41	3.8	Downregulation
2633	GBP1	3050	39.56	15.98	Downregulation
84969	TOX2	2464.95	7.99	3.23	Downregulation
144100	PLEKHA7	4820	3.14	1.27	Downregulation
84665	MYPN	5183.92	1.38	0.56	Downregulation
2674	GFRA1	9155.96	1.6	0.65	Downregulation
284	ANGPT1	3804.18	8.32	3.38	Downregulation

2825	GPR1	2168	4.97	2.02	Downregulation
3887	KRT81	1926	20.32	8.26	Downregulation
91584	PLXNA4	11890.4<br>9	30.04	12.23	Downregulation
112479	ERI2	3529	2.97	1.21	Downregulation
54507	ADAMTSL4	4001.58	8.88	3.62	Downregulation
22807	IKZF2	9512	2.03	0.83	Downregulation
2823	GPM6A	3152.19	2.2	0.9	Downregulation
857	CAV1	2556.58	100.39	41.08	Downregulation
26471	NUPR1	888.19	39.92	16.38	Downregulation
10905	MAN1A2	5388	9.8	4.04	Downregulation
117854	TRIM6	3037.16	6.86	2.83	Downregulation
4739	NEDD9	3948.91	8.5	3.51	Downregulation
101928936	LOC101928936	3974	2.88	1.19	Downregulation
5985	RFC5	2242.78	3.84	1.59	Downregulation
3875	KRT18	1458.5	204.29	84.79	Downregulation
22837	COBLL1	9374	1.18	0.49	Downregulation
27143	PALD1	4601	1.9	0.79	Downregulation
344148	NCKAP5	7611	6.13	2.55	Downregulation
1183	CLCN4	6660.17	3.94	1.64	Downregulation
201134	CEP112	3449	3.48	1.45	Downregulation
154091	SLC2A12	5599	9.13	3.81	Downregulation
139886	SPIN4	4112	1.7	0.71	Downregulation
9464	HAND2	2368	4.66	1.95	Downregulation
4921	DDR2	3172	18.33	7.68	Downregulation
112597	LINC00152	520.65	72.85	30.53	Downregulation
376267	RAB15	3284	1.98	0.83	Downregulation
57211	ADGRG6	6896	4.04	1.7	Downregulation
221079	ARL5B	3589	6.61	2.79	Downregulation
84913	ATOH8	5873	16.79	7.1	Downregulation
29915	HCFC2	5743	3.28	1.39	Downregulation
23179	RGL1	4804.59	15.54	6.59	Downregulation
10232	MSLN	2127	4.43	1.88	Downregulation
54622	ARL15	2501	4.24	1.8	Downregulation
9882	TBC1D4	6282.92	8.4	3.57	Downregulation
100507487	LOC100507487	2081	3.69	1.57	Downregulation
26353	HSPB8	2056	5.17	2.2	Downregulation
10234	LRRC17	2250	38.81	16.52	Downregulation
8424	BBOX1	1901	13.26	5.65	Downregulation
117583	PARD3B	7982.16	6.43	2.74	Downregulation
50807	ASAP1	6263.52	53.67	22.88	Downregulation
1112	FOXN3	7856	6.66	2.84	Downregulation
8322	FZD4	7394	12.25	5.23	Downregulation
144811	LACC1	4124	7.82	3.34	Downregulation
6519	SLC3A1	2353	5.94	2.54	Downregulation
100302739	PCNA-AS1	384	33.34	14.27	Downregulation
4085	MAD2L1	1453	5.11	2.19	Downregulation
154664	ABCA13	17207	1.54	0.66	Downregulation

57381	RHOJ	3619	6.53	2.8	Downregulation
2150	F2RL1	2883	3.59	1.54	Downregulation
8743	TNFSF10	1876.19	3.38	1.45	Downregulation
135228	CD109	9073.98	13.89	5.96	Downregulation
63926	ANKEF1	3829	3.61	1.55	Downregulation
51474	LIMA1	3582.78	53.4	22.93	Downregulation
83660	TLN2	11650	4.12	1.77	Downregulation
59269	HIVEP3	11269.01	2.7	1.16	Downregulation
80319	CXXC4	5104.72	4.79	2.06	Downregulation
891	CCNB1	2177	3.58	1.54	Downregulation
254228	FAM26E	3556	3.23	1.39	Downregulation
254778	C8orf46	3471	1.81	0.78	Downregulation
182	JAG1	5988	5.91	2.55	Downregulation
7049	TGFBR3	6572	1.83	0.79	Downregulation
2898	GRIK2	4716.91	4.14	1.79	Downregulation
7164	TPD52L1	2230.59	13.12	5.68	Downregulation
8821	INPP4B	4041	6.3	2.73	Downregulation
619207	SCART1	4900	1.43	0.62	Downregulation
11145	PLA2G16	1266.13	30.97	13.44	Downregulation
26586	CKAP2	3647.73	14.3	6.21	Downregulation
7048	TGFBR2	4654.16	93.94	40.86	Downregulation
9462	RASAL2	9988.05	7.7	3.35	Downregulation
6622	SNCA	3190.2	11.27	4.91	Downregulation
91614	DEPDC7	1774	6.01	2.62	Downregulation
55103	RALGPS2	7526	8.14	3.55	Downregulation
7003	TEAD1	9433	28.06	12.24	Downregulation
4810	NHS	8181	4.24	1.85	Downregulation
1755	DMBT1	7303.26	1.76	0.77	Downregulation
9020	MAP3K14	4499	9.53	4.17	Downregulation
80014	WWC2	8822	8.27	3.62	Downregulation
595135	PGM5P2	1976	7.72	3.38	Downregulation
27350	APOBEC3C	1127	14.66	6.47	Downregulation
57507	ZNF608	5645	4.21	1.86	Downregulation
3691	ITGB4	5742.31	3.44	1.52	Downregulation
8564	KMO	5266	10.25	4.54	Downregulation
824	CAPN2	3391.27	131.85	58.4	Downregulation
2305	FOXM1	3590.88	6.49	2.88	Downregulation
64750	SMURF2	3866	28.06	12.49	Downregulation
91351	DDX60L	6767.74	26.49	11.8	Downregulation
55582	KIF27	4640.54	2.89	1.29	Downregulation
80205	CHD9	11482	10.5	4.69	Downregulation
401548	SNX30	7616	5.57	2.49	Downregulation
55703	POLR3B	4238.04	4.39	1.97	Downregulation
6498	SKIL	7002.34	20.58	9.25	Downregulation
57724	EPG5	12715	8.29	3.73	Downregulation
23576	DDAH1	3997.68	37.77	17.03	Downregulation
114294	LACTB	2349.12	35.03	15.82	Downregulation

9603	NFE2L3	3720	24.83	11.22	Downregulation
8853	ASAP2	5631.4	38.2	17.27	Downregulation
166929	SGMS2	5994.86	7.83	3.54	Downregulation
340075	ARSI	3225	2.3	1.04	Downregulation
79618	HMBOX1	3175	2.41	1.09	Downregulation
4750	NEK1	5542.48	6.34	2.87	Downregulation
25805	BAMBI	1732	27.76	12.61	Downregulation
8085	MLL2	19432	17.71	8.05	Downregulation
80830	APOL6	10156	10.41	4.73	Downregulation
3832	KIF11	5101	4.29	1.95	Downregulation
79633	FAT4	16117.74	6.57	2.99	Downregulation
8706	B3GALNT1	3269	4.54	2.07	Downregulation
7052	TGM2	3930.57	685.11	312.6	Downregulation
22871	NLGN1	4947	2.76	1.26	Downregulation
50484	RRM2B	4801.91	22.09	10.1	Downregulation
116966	WDR17	7438.08	2.01	0.92	Downregulation
51203	NUSAP1	2469.47	6.68	3.06	Downregulation
134430	WDR36	6611	13.49	6.18	Downregulation
51762	RAB8B	4877	18.64	8.54	Downregulation
23180	RFTN1	3033	17.37	7.96	Downregulation
6331	SCN5A	8390.02	14.38	6.59	Downregulation
54677	CROT	3045.68	6.27	2.88	Downregulation
3157	HMGCS1	5412.59	10.42	4.79	Downregulation
6272	SORT1	7038	23.73	10.91	Downregulation
23405	DICER1	10205.94	7.58	3.49	Downregulation
440672	NUDT4P1	3802	22.14	10.2	Downregulation
4320	MMP11	2306	10.48	4.83	Downregulation
647979	NORAD	5378	98.11	45.25	Downregulation
26289	AK5	3334.46	24.24	11.18	Downregulation
8313	AXIN2	4241	2.71	1.25	Downregulation
80114	BICC1	5486	47.23	21.8	Downregulation
100996712	SRGAP2D	3046	5.89	2.72	Downregulation
374383	NCR3LG1	6382	1.71	0.79	Downregulation
11080	DNAJB4	2978.82	17.55	8.11	Downregulation
8601	RGS20	1520	13.11	6.06	Downregulation
3655	ITGA6	5615.16	10.27	4.76	Downregulation
644873	LINC01184	2977	2.5	1.16	Downregulation
11103	KRR1	3357	29.78	13.82	Downregulation
10499	NCOA2	6157	4.65	2.16	Downregulation
10085	EDIL3	4751.96	83.44	38.8	Downregulation
3953	LEPR	4636.21	13.76	6.4	Downregulation
6335	SCN9A	9771	3.59	1.67	Downregulation
11169	WDHD1	6054	1.59	0.74	Downregulation
100775107	RBM5-AS1	1386	15.27	7.11	Downregulation
4214	MAP3K1	7522	1.35	0.63	Downregulation
92691	TMEM169	3387.01	6.64	3.1	Downregulation

55568	GALNT10	5966	62.9	29.38	Downregulation
57684	ZBTB26	2008	4.04	1.89	Downregulation
27314	RAB30	9886.18	4.27	2	Downregulation
5915	RARB	2916.03	9.35	4.38	Downregulation
94241	TP53INP1	5628	12.11	5.69	Downregulation
54542	RC3H2	3941.95	10.53	4.95	Downregulation
9397	NMT2	3859.64	25.65	12.06	Downregulation
51053	GMNN	1193.19	10.44	4.91	Downregulation
79628	SH3TC2	26588	0.34	0.16	Downregulation
5581	PRKCE	5537	3.46	1.63	Downregulation
302	ANXA2	1564.34	1477.99	696.43	Downregulation
9735	KNTC1	6965	2.27	1.07	Downregulation
25758	KIAA1549L	11640	4.05	1.91	Downregulation
113146	AHNAK2	18317	14.22	6.71	Downregulation
9014	TAF1B	2280.21	6.82	3.22	Downregulation
54874	FNBP1L	4567.16	12.6	5.95	Downregulation
79776	ZFHX4	13975	5.08	2.4	Downregulation
3037	HAS2	3275	2.62	1.24	Downregulation
5565	PRKAB2	5432.52	5.26	2.49	Downregulation
115	ADCY9	7741	4.16	1.97	Downregulation
900	CCNG1	2400.1	81.19	38.51	Downregulation
79158	GNPTAB	5644	19.56	9.28	Downregulation
160518	DENND5B	9459.93	7.31	3.47	Downregulation
5205	ATP8B1	5949	20.85	9.91	Downregulation
113612	CYP2U1	4760	8.52	4.05	Downregulation
27327	TNRC6A	8438	22.63	10.77	Downregulation
440138	ALG11	2595	3.78	1.8	Downregulation
84295	PHF6	4442	10.94	5.21	Downregulation
860	RUNX2	5282.35	25.88	12.33	Downregulation
84740	AFAP1-AS1	6810	1.95	0.93	Downregulation
10873	ME3	2130.15	28.66	13.67	Downregulation
23677	SH3BP4	5193	54.99	26.25	Downregulation
9415	FADS2	3162.69	51.15	24.42	Downregulation
1787	TRDMT1	7687	1.36	0.65	Downregulation
142940	TRUB1	3412	12.93	6.18	Downregulation
148534	TMEM56	6908.68	3.7	1.77	Downregulation
1955	MEGF9	6227	52.27	25.01	Downregulation
23089	PEG10	6634.49	18.66	8.93	Downregulation
10501	SEMA6B	3961	6.43	3.08	Downregulation
9585	KIF20B	6339	3.15	1.51	Downregulation
26507	CNNM1	5988	5.15	2.47	Downregulation
54790	TET2	9741.56	7.19	3.45	Downregulation
92370	PXYLP1	3281	7.44	3.57	Downregulation
1829	DSG2	5885	33.44	16.05	Downregulation
6470	SHMT1	2469.8	6.37	3.06	Downregulation
154214	RNF217	4722.86	10.05	4.83	Downregulation
23612	PHLDA3	1855.73	39.48	18.99	Downregulation
50937	CDON	8051	3.28	1.58	Downregulation

1073	CFL2	3101.55	40.73	19.62	Downregulation
9127	P2RX6	2754	4.4	2.12	Downregulation
387882	C12orf75	1383	63.38	30.54	Downregulation
374354	NHLRC2	6409	6.76	3.26	Downregulation
25939	SAMHD1	3189	56.71	27.4	Downregulation
23092	ARHGAP26	8876	3.99	1.93	Downregulation
1612	DAPK1	5781.63	29.25	14.17	Downregulation
728730	LOC728730	2517	4.21	2.04	Downregulation
5983	RFC3	2205.62	3.24	1.57	Downregulation
401474	SAMD12	8799	3.69	1.79	Downregulation
163486	DENND1B	6706.55	5.17	2.51	Downregulation
1657	DMXL1	11694.78	5.17	2.51	Downregulation
200895	DHFRL1	3983.85	12.81	6.22	Downregulation
10783	NEK6	2627.18	43.58	21.18	Downregulation
5999	RGS4	3147.55	130.8	63.63	Downregulation
85461	TANC1	7508.55	10.19	4.96	Downregulation
55357	TBC1D2	3207.83	20.41	9.94	Downregulation
6526	SLC5A3	11623	10.81	5.27	Downregulation
134957	STXBP5	9291	16.61	8.11	Downregulation
6894	TARBP1	5130	7.76	3.79	Downregulation
10150	MBNL2	4656.12	22.03	10.76	Downregulation
2650	GCNT1	5559.61	4.36	2.13	Downregulation
4091	SMAD6	2886	4.83	2.36	Downregulation
4254	KITLG	5460	2.72	1.33	Downregulation
343450	KCNT2	5818.29	6.83	3.34	Downregulation
2122	MECOM	5374.56	4.74	2.32	Downregulation
6586	SLIT3	9727	5	2.45	Downregulation
54899	PXK	2869.21	31.18	15.28	Downregulation
79674	VEPH1	3902.4	21.15	10.37	Downregulation
57574	4-Mar	4466	5.18	2.54	Downregulation
100302736	TMED7-TICAM2	3655	4.18	2.05	Downregulation
9652	TTC37	5704	27.56	13.53	Downregulation
3177	SLC29A2	2608.13	5.31	2.61	Downregulation
100288142	NBPF20	18450	3.17	1.56	Downregulation
79006	METRN	1109	15.05	7.42	Downregulation
4919	ROR1	4250.54	1.58	0.78	Downregulation
4133	MAP2	5801.29	10.39	5.13	Downregulation
57102	C12orf4	3868	7.53	3.72	Downregulation
22847	ZNF507	7693.56	3.64	1.8	Downregulation
23175	LPIN1	4312.52	17.7	8.76	Downregulation
26136	TES	2743.36	25.26	12.51	Downregulation
101927755	LOC101927755	1521.15	7.73	3.83	Downregulation
7145	TNS1	10036.05	14.02	6.95	Downregulation
3856	KRT8	1907.63	203.85	101.09	Downregulation
5793	PTPRG	6648	5.57	2.77	Downregulation
10602	CDC42EP3	5165.49	60.68	30.2	Downregulation

100505385	IQCJ-SCHIP1	2384	6.65	3.32	Downregulation
9262	STK17B	5324	16.92	8.45	Downregulation
100861518	P4HA2-AS1	836	10.87	5.43	Downregulation
8850	KAT2B	4824	13.72	6.86	Downregulation
55196	KIAA1551	6246	9.18	4.59	Downregulation
55785	FGD6	9288	7.56	3.78	Downregulation
